# A Preliminary Randomized Double Blind Placebo-Controlled Trial of Intravenous Immunoglobulin for Japanese Encephalitis in Nepal

**DOI:** 10.1371/journal.pone.0122608

**Published:** 2015-04-17

**Authors:** Ajit Rayamajhi, Sam Nightingale, Nisha Keshary Bhatta, Rupa Singh, Elizabeth Ledger, Krishna Prasad Bista, Penny Lewthwaite, Chandeshwar Mahaseth, Lance Turtle, Jaimie Sue Robinson, Sareen Elizabeth Galbraith, Malgorzata Wnek, Barbara Wilmot Johnson, Brian Faragher, Michael John Griffiths, Tom Solomon

**Affiliations:** 1 Institute of Infection and Global Health, University of Liverpool, Liverpool, United Kingdom; 2 National Academy of Medical Sciences, Kathmandu, Nepal; 3 Kanti Children’s Hospital, Kathmandu, Nepal; 4 BP Koirala Institute of Health Sciences, Dharan, Nepal; 5 Centers for Disease Control and Prevention, Division of Vector-Borne Diseases, Arbovirus Diseases Branch Diagnostic & Reference Laboratory, Fort Collins, Colorado, United States of America; 6 Institute for Health and Wellbeing, Leeds Metropolitan University, Leeds, United Kingdom; 7 Liverpool School of Tropical Medicine, Liverpool, United Kingdom; 8 Alder Hey Children’s National Health Service Foundation Trust, Liverpool, United Kingdom; 9 The Walton Centre National Health Service Foundation Trust, Liverpool, United Kingdom; 10 National Consortium for Zoonosis Research, Liverpool, United Kingdom; 11 National Institute for Health Research—Health Protection Research Unit in Emerging and Zoonotic Infections, Liverpool, United Kingdom; University of Ottawa, CANADA

## Abstract

**Background:**

Japanese encephalitis (JE) virus (JEV) is a mosquito-borne flavivirus found across Asia that is closely related to West Nile virus. There is no known antiviral treatment for any flavivirus. Results from *in vitro* studies and animal models suggest intravenous immunoglobulin (IVIG) containing virus-specific neutralizing antibody may be effective in improving outcome in viral encephalitis. IVIG’s anti-inflammatory properties may also be beneficial.

**Methodology/Principal Findings:**

We performed a pilot feasibility randomized double-blind placebo-controlled trial of IVIG containing anti-JEV neutralizing antibody (ImmunoRel, 400mg/kg/day for 5 days) in children with suspected JE at two sites in Nepal; we also examined the effect on serum neutralizing antibody titre and cytokine profiles. 22 children were recruited, 13 of whom had confirmed JE; 11 received IVIG and 11 placebo, with no protocol violations. One child (IVIG group) died during treatment and two (placebo) subsequently following hospital discharge. Overall, there was no difference in outcome between treatment groups at discharge or follow up. Passive transfer of anti-JEV antibody was seen in JEV negative children. JEV positive children treated with IVIG had JEV-specific neutralizing antibody titres approximately 16 times higher than those treated with placebo (p=0.2), which was more than could be explained by passive transfer alone. IL-4 and IL-6 were higher in the IVIG group.

**Conclusions/Significance:**

A trial of IVIG for JE in Nepal is feasible. IVIG may augment the development of neutralizing antibodies in JEV positive patients. IVIG appears an appealing option for JE treatment that warrants further study.

**Trial Registration:**

ClinicalTrials.gov NCT01856205

## Introduction

Japanese encephalitis virus (JEV) infection is the most important cause of epidemic encephalitis worldwide, with over 60,000 cases annually [1]. JEV is found in Southeast Asia, China, the Pacific Rim and the Asian subcontinent, and its geographical range is expanding [2]. JEV is a small single-stranded positive-sense RNA flavivirus, closely related to West Nile virus (WNV) that is transmitted between its natural bird and pig hosts by *Culex tritaeniorhynchus* and other mosquitoes. JEV transmission occurs mainly in rural areas where rice crops are cultivated and where the *Culex* mosquito favours sources of stagnant water in which to breed. Most people living in rural Asia are infected during childhood, but few of those infected become symptomatic. Those that do develop symptoms, usually present with severe meningo-encephalitis and seizures [3]. Around 20–30% of patients with neuroinvasive JEV infection die, and half of the survivors have severe neurological sequelae. This imposes a huge socioeconomic burden in the poor rural settings where JEV is found [4].

Although vaccines against JEV have become more widely used in recent years, the animal reservoir cannot be eradicated, so JEV remains a threat. The virus has continued to spread and at present there is no established treatment for JEV, or other related flaviviruses such as WNV.

The pathogenesis of Japanese encephalitis (JE) is believed to involve a combination of viral cytopathology and immunopathology [5–8]; previous attempts to develop treatment have explored both of these. After entering the body through the bite of an infected mosquito, JEV amplifies in the dermal tissues and lymph nodes leading to viremia. Virions are thought to then bind to the vascular endothelial surface within the CNS, be internalized by endocytosis and transferred across the endothelial cells [2]. In the brain, JE is characterized as perivascular inflammation with recruitment of macrophages, neutrophils and lymphocytes [9–11]. The thalamus, basal ganglia, midbrain and anterior horns cells of the spinal cord are particularly affected [12, 13]. Viral antigen is predominantly in neurons although microglial cells, astrocytes and vascular endothelial cells are also infected. JEV is thought to cause neuronal cell death in two ways; firstly, direct neuronal killing [14, 15], whereby viral multiplication within neuronal cells leads to cell death; secondly, indirect killing, whereby the over activation of microglia, astrocytes and recruited macrophages [16] leads to release of excess proinflammatory cytokines such as interleukin 6 (IL-6), TNF-α, and RANTES (regulated upon activation, normal T cell expressed and secreted), which are thought to damage neuronal cells, and increase the permeability of the blood brain barrier and promoting massive leukocyte migration into the brain and further neuronal cell death [17].

The role of corticosteroids in the treatment of JE was examined in a randomized-placebo controlled trial in Thailand; although dexamethasone caused a reduction in cerebrospinal (CSF) opening pressures and CSF white cell counts, there was no overall benefit in terms of outcome [18]. Interferon-α (IFN-α) is produced as part of the innate response to JEV infection, and has antiviral activity against JEV; but a placebo-controlled trial in Vietnam found it did not improve outcome [19]. Oral ribavirin also proved to be unhelpful in a controlled trial in India [20]. Intravenous immunoglobulin (IVIG) has been postulated as a potential treatment for flavivirus encephalitis caused by JEV and WNV, on account of its antiviral and anti-inflammatory properties. It has been used on a compassionate basis, but has not been assessed in a randomized trial for either virus [17, 21]. IVIG is postulated to act in two ways; IVIG produced in countries where flaviviruses are endemic contains high titers of specific neutralizing antibody, because most of the population have been exposed to the virus, and thus have antibodies [22]. In addition IVIG has non-specific anti-inflammatory properties, particularly through the suppression of pro-inflammatory cytokines. However IVIG is more difficult to administer than intravenous IFN-α, intravenous corticosteroids or oral ribavirin [23, 24]; it is delivered using a syringe driver, and must be started at a low infusion rate, being increased over time if it is well tolerated. Where-as the previous trial using intravenous agents were conducted in settings with highly developed research infrastructure in Thailand and Vietnam, Nepal has no such establishments. We therefore conducted a preliminary randomized placebo-controlled trial of IVIG treatment in children with suspected JE in Nepal, primarily to assess feasibility. We also used the opportunity to begin examining changes in immune parameters associated with such treatment.

## Methods

### Ethics Statement

The study protocol was approved by the Nepal Health Research Council, and the ethics committees of BPKIHS, Kanti Children’s Hospital and the Liverpool School of Tropical Medicine. Informed written consent was obtained for all children from parent or legal guardian. Written consent was also obtained from JEV vaccinated individual.

### IVIG selection

JEV-specific neutralizing antibody titre was measured in pharmaceutical grade IVIG obtained from four different suppliers from areas endemic for JEV [Bharat serum, India; Hualan Biological Engineering Inc., China; Sichuan Yuanda Shuyang Pharmaceutical Co., China; Reliance Biopharmaceutical, India] by 50% plaque reduction neutralization titres (PRNT_50_) against wild type JEV (strain P3) infecting a standard culture of Vero cells. IVIG from a low JEV prevalence region (Vigam, Bio Products Laboratory, USA) and a sample of serum from a JEV vaccinated individual were tested as controls. The test was repeated three times. IVIG produced by Reliance Biopharmaceuticals Pvt. Ltd. in India and manufactured in China from blood donors in an endemic area known to have high levels of JEV seroprevalence had the highest mean JEV PRNT_50_, titre and was selected for use in this trial. In order to confirm the JEV-specific neutralizing antibody titre as well as characterise background immunity to other flaviviruses known to circulate in Asia, the IVIG was tested by dengue virus (DENV), tick-borne encephalitis virus (using Powassan virus), and WNV IgM and IgG antibody capture ELISA and PRNT_90_ at the US Centers for Disease Control and Prevention (CDC) Division of Vector-Borne Diseases [25–28]. The lower limit of quantitation (LLOQ) by PRNT90 was 1:10 serum dilution.

### Patients

Patients were recruited from two centers in Nepal: Kanti Children’s Hospital in Kathmandu, which is the main pediatric center in Nepal, and the Paediatric Department at BP Koirala Institute of Health Sciences (BPKIHS), a large regional hospital in the town of Dharan in the eastern lowland Terai area of Nepal. The JEV mosquito vector is uncommon in Kathmandu due to the elevation of 1350 meters (4400 feet) and most cases at Kanti Children’s Hospital were referred to this center from the central lowland Terai area where the disease is endemic.

During the monsoon period from May to June 2009 we recruited children aged between 1 and 14 years who had clinically diagnosed acute encephalitis syndrome (AES). On the basis of results from previous studies [19, 29] AES was diagnosed clinically in children who had a history of fever that lasted less than 14 days, altered consciousness [Glasgow coma score (GCS) <15] with or without a history of seizures, and consistent cerebrospinal fluid (CSF) findings. The CSF criteria for a clinical diagnosis of encephalitis were a white cell count less than 1000 cells/mm^3^ with no organisms on Gram stain and a CSF:plasma glucose ratio > 40% [3]. Children with a single seizure lasting less than 15 minutes with full recovery of consciousness within 60 minutes were assumed to have had a febrile convulsion and excluded. We also excluded those with a positive blood slide or rapid antigen test for *Plasmodium falciparum* parasites and those referred from a peripheral health center who had clinical and/or laboratory features suggestive of bacterial meningitis where antibiotic treatment had already been commenced. Children with a GCS < 3 (out of 15), who were receiving artificial ventilation without signs of spontaneous respiration, and with absent oculocephalic reflex were excluded, as prior studies have shown an extremely low chance of meaningful recovery [3, 30].

### Procedures

We randomly allocated patients to treatment with IVIG or placebo. Randomization was stratified by each centre using a variable block size of 4, 6 or 8, selected on a random basis similar to those schemes used previously [10]. The randomization code for each patient was kept in a sealed envelope in a locked cupboard on a separate ward (Randomization schedule is provided as S1 Table). Once a patient was enrolled, technicians who were not otherwise connected to the study opened the envelope, drew up the study drug, covered the syringe in opaque tape (because of subtle differences in colour and viscosity of the two substances) and delivered it to the ward, where the nurses gave the treatment. Children received either saline or intravenous immunoglobulin (IVIG) [ImmunoRel (batch 20081217)] at a dose of 400mg/kg/day for 5 days or an equivalent volume of 0.9% normal saline. The infusion was started at an initial rate of 0.01 to 0.02 ml/kg body weight/minute and if well tolerated the rate was gradually increased over 30 to 60 minutes to a maximum rate of 0.08 ml/kg body weight/minute. Empty vials were concealed in paper bags and disposed of. Hence, all investigators, care providers and participants were blinded of the study drug. A second sealed envelope was kept at the back of each patient’s inpatient notes in case a physician urgently needed to know which drug a patient had received. We took a detailed history and a member of the study team did daily clinical and neurological examinations (or more frequently if needed) until death or discharge. The primary aim was to assess the feasibility of a multicentre placebo-controlled RCT of IVIG in this resource-poor setting. Secondary outcomes were death or neurological sequelae at discharge or follow up and, adverse events. In particular side effects monitored were infusion site reaction, diarrhoea, rise in blood pressure and change in urinary output daily from first day of commencing treatment which was on first day of hospital admission until death or discharge which was on average on eighth day. We also wanted to examine any difference in anti-JEV antibody titres between children treated and untreated with IVIG. Adverse events were graded using WHO recommendations and reported to an independent data safety monitoring committee. Information was recorded on standardized proformas. Computer tomography scans were performed as indicated at the discretion of the admitting physician. There were no onsite facilities for magnetic resonance imaging. Patients with prolonged or repeated seizures, respiratory difficulties or severely reduced consciousness, were admitted to a paediatric intensive care unit, which had facilities to intubate and ventilate. Mannitol and dexamethasone were given at the discretion of the admitting physician, and suspected septicaemia was treated with broad-spectrum antibiotics. Patients were assessed at discharge and 3–6 month follow-up for disability in a range of activities using the Liverpool Outcome Score [31] and a standardized neurological assessment. The Liverpool Outcome Score assesses speech, communication, feeding, ability to be left alone, behaviour, recognition, school or working, seizures, dressing, bladder/bowel control, hearing, sitting, standing, walking and upper limb dexterity. Children were classified as I if the child died; II, if there were severe sequelae causing impairment of function likely to make the child dependent on others; III, if there were moderate sequelae only mildly affecting function, probably compatible with independent living, IV, if there were minor sequelae with mild effects on function or personality change or on medication; V, if there was complete recovery which included normal examination (http://www.liv.ac.uk/infection-and-global-health/research/brain-infections-group/education.). Children who did not return for follow-up were reminded by letter or telephone, as available. If they were unable to return to the hospital, they were examined at home. At follow-up, we took particular note of seizures, progress at school, and changes in personality. The trial protocol is available as S1 Protocol and online (http://clinicaltrials.gov/show/NCT01856205). CONSORT checklist is provided as S1 Checklist CONSORT.

### Diagnostic and Pathogenesis studies

On admission patients had routine blood tests (typically; a full blood count and white cell differential, glucose, urea and electrolytes) and a blood film or rapid antigen test for *Plasmodium falciparum*. For diagnostic and pathogenesis studies, we took blood prior to treatment (IVIG or placebo) and 1 hour after the completion of infusion of the 5^th^ (final) dose. If a child required further blood tests for clinical reasons, extra samples were collected. Serum was separated by centrifuge, frozen at -20^°^C; aliquots were made and one aliquot went to the designated laboratory in Nepal for testing; the other aliquots were transported on dry ice to the Institute of Infection and Global Health, Liverpool, UK, and subsequently to CDC Division of Vector-Borne Diseases in Fort Collins, Colorado, USA.

On admission, we did a lumbar puncture with patients in the lateral position, and if the patient was calm the opening pressure was recorded using a cerebrospinal-fluid manometer. The lumbar puncture was delayed in patients who were convulsing, or those with clinical signs of raised intracranial pressure [32]. CSF samples were examined for cell count and differential, protein, glucose and Gram stain. Further CSF and serum samples were frozen on site at -20**°**C, and subsequently transported for further investigations, as above.

To diagnose JE locally, anti-JEV immunoglobulin-M (IgM) antibodies were measured in all serum samples on admission and day 7 of admission, and in all CSF samples by enzyme-linked immunosorbent assay (ELISA) using the AFRIMS JE MAC IgM ELISA as part of the WHO acute encephalitis syndrome surveillance programme; these samples were measured in batches at the National Public Health Laboratories in Kathmandu, and so the results were not available at the time of randomisation. Classification of the patients as JE negative or positive was based on the results of this initial JEV IgM ELISA testing in Nepal, confirmed at CDC, USA, as below.

Confirmatory testing was subsequently performed at the CDC Arboviral Diseases Branch diagnostic laboratory using the following testing algorithm. The last serum sample collected from each patient was tested by JEV and DENV IgM capture ELISA. Positive or equivocal results were confirmed by JEV and DENV PRNT_90_, with a 4-fold or greater difference in titre interpreted to be virus specific. If the last sample was positive or equivocal for JEV and/or DENV IgM, all samples for that case were tested. However, if the last serum sample from a patient was collected at less than 7 days post onset of disease and had an IgM ELISA negative result, the CSF from that patient was also tested by JEV and DENV IgM ELISA.

In addition, if the last serum sample collected from a case had a negative JEV and DENV IgM ELISA result and was collected <10 days from illness onset, CSF was tested for the presence of JEV ribonucleic acid (RNA) by two real-time reverse transcription-polymerase chain reaction (RT-PCR) assays, one designed in-house at CDC (JEV239F: GGCTCTTATCACGTTCTTCAAGTTT; JEV344R: ACTAGTAAGATGTTTCATTGCCACACTCT; JEV269probe: ATTAGCCCCGACCAAGGCGCTTT) and other developed by Parida et al. [33]. Both assays had sensitivities of 0.001 equivalent plaque forming units for SA-14-14-2 reference viral RNA [33]. Viral RNA was extracted from 100μl of CSF, eluted in 50 μl using the QIAamp Viral RNA Mini Kit (QIAGEN, Valencia, CA); 20 μl RNA template was used in a 50μl reaction using the iScript One-Step RT-PCR kit for Probes (BioRad, CA).

To study in more detail the effect of IVIG on the neutralising antibody titres, JEV PRNT_50_ was done on serum from all patients at pre-treatment (immediately prior to the first dose), and post-treatment (1hour after the 5^th^ dose).

Interleukin (IL) -6 and IL-4 cytokine measurements were undertaken on aliquots of IVIG and on patients’ serum pre-treatment (before first dose) and post-treatment (1 hour after 5^th^ dose), using ELISA kits following the manufacture’s protocol (Bender MedSystems GmBh, Wien, Austria). Samples were measured in duplicate (50ul) at each time-point. Quality control was based on concurrent measurement of negative and positive controls supplied in the kits.

### Sample size

The primary aim of this study was to examine feasibility, through studying approximately 20 patients across two sites. In addition, we wished to determine if there were any adverse effects of IVIG or any differences in presence of anti-JEV antibody titres following administration of IVIG and placebo. To our knowledge, there are no previous human clinical trials of IVIG among JE patients to guide sample size. Similarly, they have been no toxicity studies of IVIG in animals with JE. However, there are data on change anti-JEV titre associated with improved outcome in mice given immune splenocytes. In the latter study, mice given immune splenocytes generated a mean anti-JEV titre of 400, whereas mice given naïve splenocytes exhibited a mean anti-JEV titre of 40. Based on these data, using a significance of 0.95 and a power of 0.8, mean titres of 400 and 40 and a standard deviation of 400 and 40 for anti-JEV antibody titres in IVIG exposed and non-exposed respectively, we required 10 subjects in each arm. To allow for loss to follow up of 10–20% we aimed to recruit 22–24 patients. This small sample size would also allow us to look for any adverse events associated with treatment, accepting that for a true dose-limiting toxicity of 0.05 and 11 JEV subjects receiving IVIG, the probability of observing at least one dose-limiting toxicity associated with IVIG was only 0.4312.

### Statistical analysis

Patient clinical features and laboratory parameters were compared between treatment groups (IVIG or placebo). Difference in plaque reduction anti-JEV antibody neutralizing titres (PRNTs) pre and post treatment were also compared between treatment groups (IVIG or placebo). Similarly, the difference in cytokine abundance pre and post treatment (separately for IL4 and IL6) were compared between treatment groups. Normally distributed data were compared using Student’s t-test. Non-normally distributed data were compared using the Wilcoxon-Mann-Whitney test. Differences between proportions were tested using the Fisher’s exact test. To examine whether JE antibody status of the participant prior to treatment was a source of variation in difference in antibody titres or cytokine abundances following treatment, a 2 way ANOVA was undertaken, where treatment (IVIG or placebo) was the column factor and JE status (anti-JEV antibody positive or negative) was the row factor. Prism ver.6.04 (Graph Pad Software, Inc) was used for statistical analyses and graph generation.

## Results

### Patients

**Fig 1:**
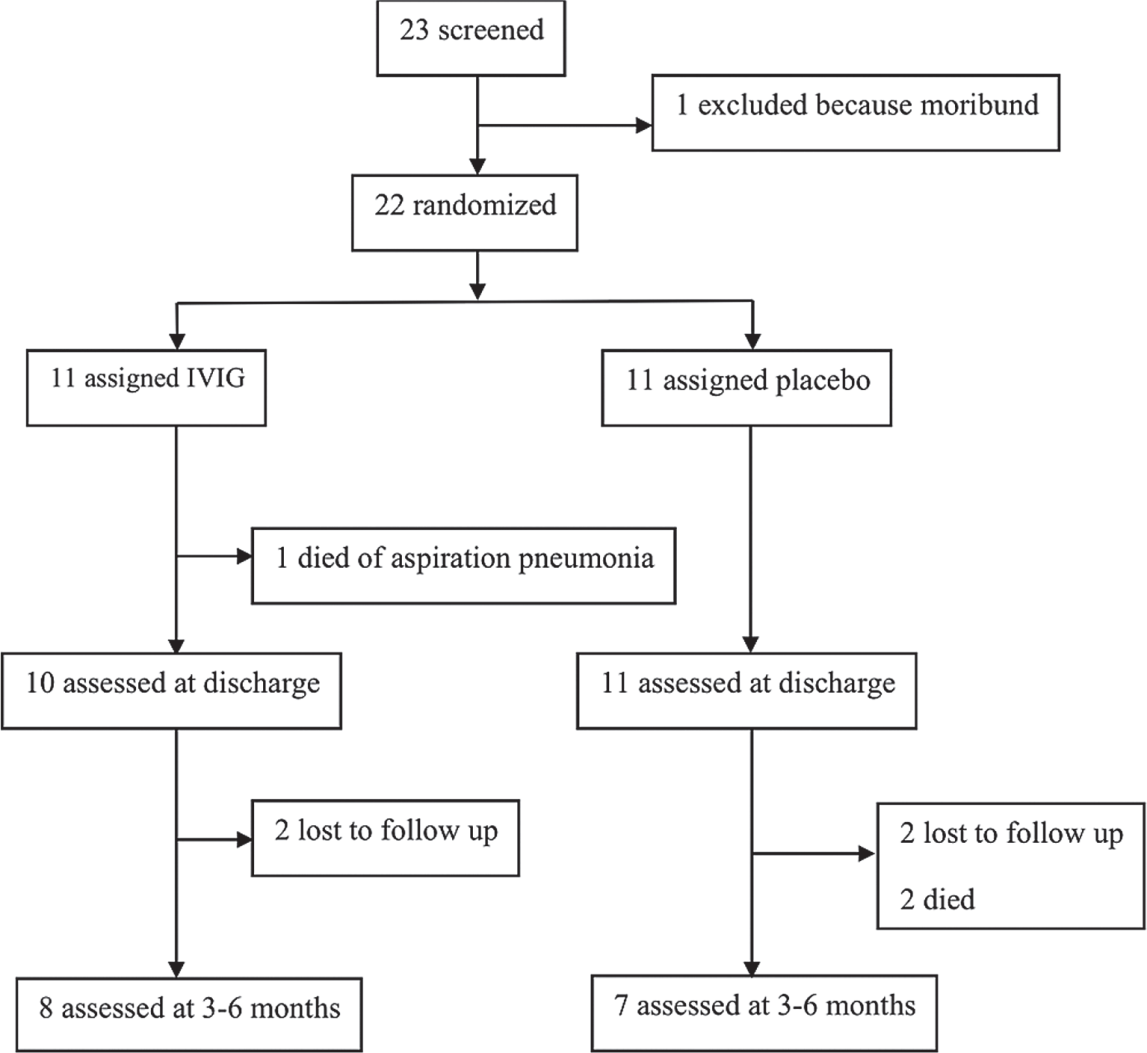
Flow diagram of study participants’ recruitment and follow-up. All children enrolled, fitting the trial criteria, who were alive at discharge were attempted to be followed-up (n = 21). Twenty-one families were successfully contacted. Among these families, two children had died.

Between May and September 2009, 22 (96%) children of 23 screened met the entry criteria; 12 in Kanti Children’s Hospital in Kathmandu and 10 in BPKIHS in Dharan. One child was not enrolled in the trial as he was moribund and met criteria for exclusion. Eleven (50%) received IVIG and 11 (50%) placebo (Fig 1). Twenty-two patients were randomized and there were no breaches of randomization protocol. None of the children were reported to have been vaccinated against JE. Seven (64%) patients in the IVIG group and 6 (55%) in the placebo group were classified as acute JEV infections by JEV/DENV IgM ELISA testing**.** The initial JE diagnostic classification for all patients in Nepal was confirmed by the testing at the CDC, USA. Seven out of 10 (70%) were positive for JEV in BPKIHS and 6 of 12 (50%) in Kanti Children’s Hospital. One child (placebo group) had evidence of a previous flavivirus infection. One patient with an undetermined status after local testing was found to be JEV negative by confirmatory testing at CDC; otherwise there was complete concordance between the test results in Nepal and those at CDC. All CSF tested were negative for JEV RNA by real time RT-PCR.

The baseline characteristics of the two groups were comparable (Table 1). The median age was 5 and 7 years for treatment and placebo groups respectively (not significantly different). Most patients came from rural areas, and arrived after approximately 5 days of illness. Median GCS on admission was 8 (range 5–15), and the majority of patents had seizures as part of the presenting syndrome. All concurrently received antibiotics, most commonly cephalosporins. Four patients received dexamethasone alongside study drug; all 4 were in the placebo arm. One child received only 1 dose, 2 children had 2 doses each, and 1 child had daily dexamethasone for 5 days (Raw data of patients’ characteristics is available as S2 Table).

**Table 1:** Baseline characteristics of trial participants.

Parameters	No.	IVIG (n = 11)		No.	Placebo (n = 11)	*P*
Age (years)	11	5 (1–11)		11	7 (1.3–12)	NS.
Male	11	5 (.45)		11	7 (.64)	NS.
Living in rural area	11	11 (1)		11	9 (.82)	NS.
Fever duration (days)	9	5 (2–12)		11	5 (3–10)	NS.
Illness duration (days)	10	5 (4–13)		11	6 (3–13)	NS.
Altered sensorium	11	10 (.91)		11	9 (.81)	NS.
New onset seizures	11	10 (.91)		11	11 (1)	NS.
Glasgow Coma Scale (3–15) on admission	11	8 (5–15)		11	8 (5–15)	NS.
Temperature (°C)	10	38.3 (36.7–40)		10	38.9 (36.7–40)	NS.
Heart rate (beats/minute)	10	95 (68–140)		11	104 (84–130)	NS.
Resp. rate (breaths/minute)	10	33.5 (22–56)		11	28 (22–40)	NS.
Neck stiffness present	11	4 (.36)		11	4 (.36)	NS.
Kernig's sign present	11	1 (.09)		10	2 (.18)	NS.
Abnormal limb tone	8	5 (.63)		9	3 (.3)	NS.
Abnormal posturing	9	2 (.22)		11	0 (0)	NS.
Positive anti-JEV IgM	11	7 (.64)		11	6 (.55)	NS.
Haemoglobin (g/dl)	9	12.1 (9.8–13.8)		10	11.8 (8.2–14.3)	NS.
White cell count (WCC)—x10^9^/L	9	10.8 (5.2–25.4)		11	13.9 (4.2–18.9)	NS.
Polymorphs (%)	9	66 (30–85)		11	68 (30–90)	NS.
Lymphocytes (%)	9	31 (12–70)		11	30 (10–70)	NS.
Platelets (x10^9^/L)	7	270 (120–659)		9	200 (88–362)	NS.
Glucose (mg/dl)	11	100 (80–160)		11	85 (66–170)	0.03
Urea (mg/dl)	7	22.8 (15–35)		9	27.0 (18–58)	NS.
Creatinine (mg/dl)	7	0.9 (0.4–2.4)		9	1 (0.5–2.2)	NS.
Cerebrospinal fluid (CSF) WCC-cells/mm^3^	11	35 (0–125)		11	30 (0–300)	NS.
CSF polymorphs (%)	11	10 (0–60)		11	10 (0–95)	NS.
CSF lymphocytes (%)	11	70 (0–100)		11	40 (0–100)	NS.
CSF protein (mg/dl)	11	39 (28–100)		10	27 (7–68)	0.01
CSF glucose (mg/dl)	11	70 (48–90)		10	61.5 (45–81)	NS.
CSF/blood glucose ratio	11	62 (50–82)		10	68.5 (46–100)	NS.
Mannitol (given)	11	3 (.27)		11	7 (.64)	NS.
Dexamethasone	9	0 (0)		11	4 (.36)	NS.
Quinine	11	5 (.45)		11	5 (.45)	NS.
Aciclovir	11	1 (.91)		11	2 (.18)	NS.
Chloramphenicol	11	1 (.91)		11	0 (0)	NS.
Cephalosporin	11	10 (.91)		11	10 (.91)	NS.
Phenytoin	11	9 (.82)		11	8 (.73)	NS.
Phenobarbitone	11	3 (.27)		11	2 (.18)	NS.

Data presented as number of patients (proportion) or median (range). No.—number of patients in the clinical group where data for the parameter was available. *P—*P value for Fisher’s Exact or Mann Whitney U test between groups.

NS.—not significant.

The adverse and serious adverse events, according to patients’ JEV status, and whether they received IVIG or placebo is presented in Table 2; there were no significant differences in frequency of adverse events between IVIG and placebo. There were no suspected unexpected serious adverse reactions (SUSARs). Raw adverse events data is available in S3 Table.

**Table 2:** Summary of adverse events.

Symptoms	IVIG			Placebo			IVIG vs. Placebo	IVIG vs. Placebo
	JE (n = 7)	Non-JE (n = 4)	Total(n = 11)	JE (n = 6)	Non-JE (n = 5)	Total(n = 11)	*P*- value	OR(95% CI)
Fever	2 (.29)	2 (0.5)	4 (.36)	1 (.17)	1 (0.2)	2 (.18)	0.64	2.6 (0.3–29)
Dyspnoea	1(.14)	2 (0.5)	3 (.27)	1 (.17)	1 (0.2)	2 (.18)	1	1.7 (0.2–20.1)
Vomiting	0 (0)	0 (0)	0 (0)	1 (.17)	0 (0)	1 (0.9)	1	0 (0–18.7)
Irritable	1(.14)	1 (.25)	2 (.18)	2 (.33)	0 (0)	2 (.18)	1	1 (0.07–13.5)
Non-urticarial skin rash	0 (0)	0 (0)	0 (0)	1 (.17)	0 (0)	1 (0.9)	1	0 (0–18.7)
Hypotension*	1(.14)	0 (0)	1 (0.9)	1 (.17)	0 (0)	1 (0.9)	1	1 (0–43.7)
Melena*	1(.14)	0 (0)	1 (0.9)	0 (0)	1 (0.2)	1 (0.9)	1	1 (0–43.7)
Death*	0 (0)	1(.14)	1 (0.9)	0 (0)	0 (0)	0 (0)	1	NA

Data are number of patients (proportion).

* Serious adverse events. NA: not applicable

### Intravenous immunoglobulin

**Fig 2:**
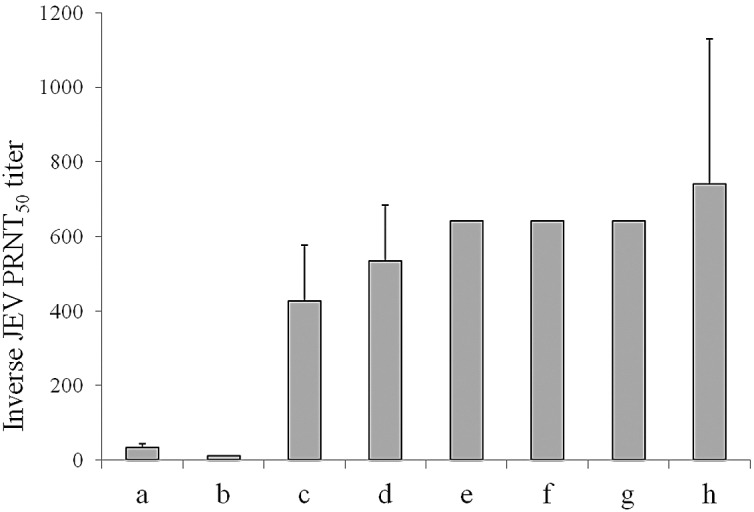
Anti-JEV neutralizing antibody in commercially available IVIG. Mean and standard deviation of reciprocal 50% plaque reduction neutralization titres (PRNT_50_) in vero cells using P3 wild type strain of JEV are shown for a: Serum control from JEV vaccinated individual, b: Vigam (USA), c: Bharat (India) batch 1, d: Hualan (China) batch 1, e: Bharat (India) batch 2, f: Hualan (China) batch 2, g: Sichuan (China), h: Reliance (India).

All IVIG preparations produced in JEV endemic regions had anti-JEV PRNT_50_ titres, ranging from 1:320 to 1:640 (Fig 2). Control IVIG from a non-JEV endemic area (Vigam, USA) showed minimal PRNT_50_ titres of 1:10; lower than serum from a JEV vaccinated individual who had a titre of 1:40. ImmunoRel IVIG produced by Reliance Biopharmaceutical (India) had the highest anti-JEV PRNT_50_ titre, and was chosen for treatment in this study. This ImmunoRel IVIG showed low PRNT_50_ titres ≤1:20 against DENV, WNV and Powassan viruses.

### Outcomes

One patient, who received the study drug, died of aspiration pneumonia. He was a 19 month old boy admitted with a 13 day history of high grade fever, repeated generalized tonic clonic seizure, fast breathing and altered sensorium. He had not been immunized against JE. His Glasgow Coma Score 10/15, pulse 140 per minute, respiratory rate 56 breaths per minute, axillary temperature 38.9^°^C, and he had diffuse wheeze in both the lung fields. On neurological examination he had a right sided hemiplegia. Investigations revealed a peripheral blood total white cell count of 25.4x10^9^ cells/L (polymorphs 66%, lymphocytes 31%), serum creatinine 0.6 mg/dl, serum sodium 143 mmol/L, serum potassium 4.5 mmol/L. His CSF was clear and colourless with 10 cells/mm^3^ (polymorphs 40%, lymphocytes 66%), protein 80mg/dl, glucose 48mg/dl (blood glucose 87 mg/dl). He was treated with intravenous ceftrioxone 100mg/kg/day for two days, intravenous midazolam at 0.2mg/kg/dose for two doses, intravenous phenytoin at 20mg/kg loading dose, followed by 7 mg/kg/day of maintenance dose, intravenous 20% mannitol at 5ml/kg single dose and maintenance intravenous fluids, but died on the fourteenth day of illness. Un-blinding after death revealed he had received IVIG (2 grams per kilogram body weight) at 0.01mL/Kg/minute on the first and second day. He was subsequently found to be JEV IgM negative. The investigating member of the Data Safety & Monitoring Committee confirmed that the child died of aspiration pneumonia due to repeated uncontrolled seizures, unrelated to the study drug.

The remaining 21 patients received the full 5 days of treatment. At hospital discharge, the commonest Liverpool outcome score was II (major sequelae) in both IVIG and the placebo groups [7/11 (64%) and 8/11 (73%) patients per group respectively]. When all participants are included at 3–6 month follow-up; 6 out of 11 (45%) exhibited complete recovery (no neurological sequelae) in the IVIG group compared to 2 out of 11 (18%) in the placebo group (Table 3). There was no significant difference in the proportion of patients exhibiting complete recovery between the two groups when analyzed by intention-to-treat either at hospital discharge or at follow-up [p = 1 and 0.36 respectively (Table 3)].

**Table 3:** Outcome for trial participants.

**Outcome at discharge**	**IVIG (n = 11)**	**Placebo (n = 11)**	***P-*value**	**OR (95% CI)**
Median duration of hospital stay (days)	13 (9–21)	12 (6–18)	0.59	-
Median Glasgow coma score	14 (3–15)	14 (7–15)	0.53	-
Number with complete recovery (LOS V)	1 (.09)	1 (.09)	1	1 (0–43.7)
Number with minor sequelae (LOS IV)	0	1 (.09)	1	0 (0–18.7)
Number with moderate sequelae (LOS III)	2 (.18)	1 (.09)	1	2.2(0.1–74.9)
Number with severe sequelae (LOS II)	7 (.64)	8 (.73)	1	0.7 (0.07–5.5)
Number that died (LOS I)	1 (.09)	0	1	NC
				
**Outcome at 3–6 months**	**IVIG (n = 11)**	**Placebo (n = 11)**	***P*-value**	**OR (95% CI)**
Lost to follow-up	2 (.18)	2 (.18)	1	1 (0.07–13.5)
Number with complete recovery (LOS V)	5(.45)	2 (.18)	0.36	3.8 (0.4–41.8)
Number with minor sequelae (LOS IV)	0	3 (.27)	0.21	0 (0–2.2)
Number with moderate sequelae (LOS III)	1 (.09)	0 (0)	1	NC
Number with severe sequelae (LOS II)	2 (.18)	2 (.18)	1	1 (0.07–13.5)
Number that died (LOS I)	1 (.09)	2 (.18)	1	0.45 (0.01–8.4)

Data are number of patients (proportion) or median (range). LOS = Liverpool Outcome Score (LOS): 1—died; 2—severe sequelae; 3—moderate sequelae; 4—minor sequelae; 5—full recovery. NC: not calculable

### Neutralizing antibodies

**Fig 3:**
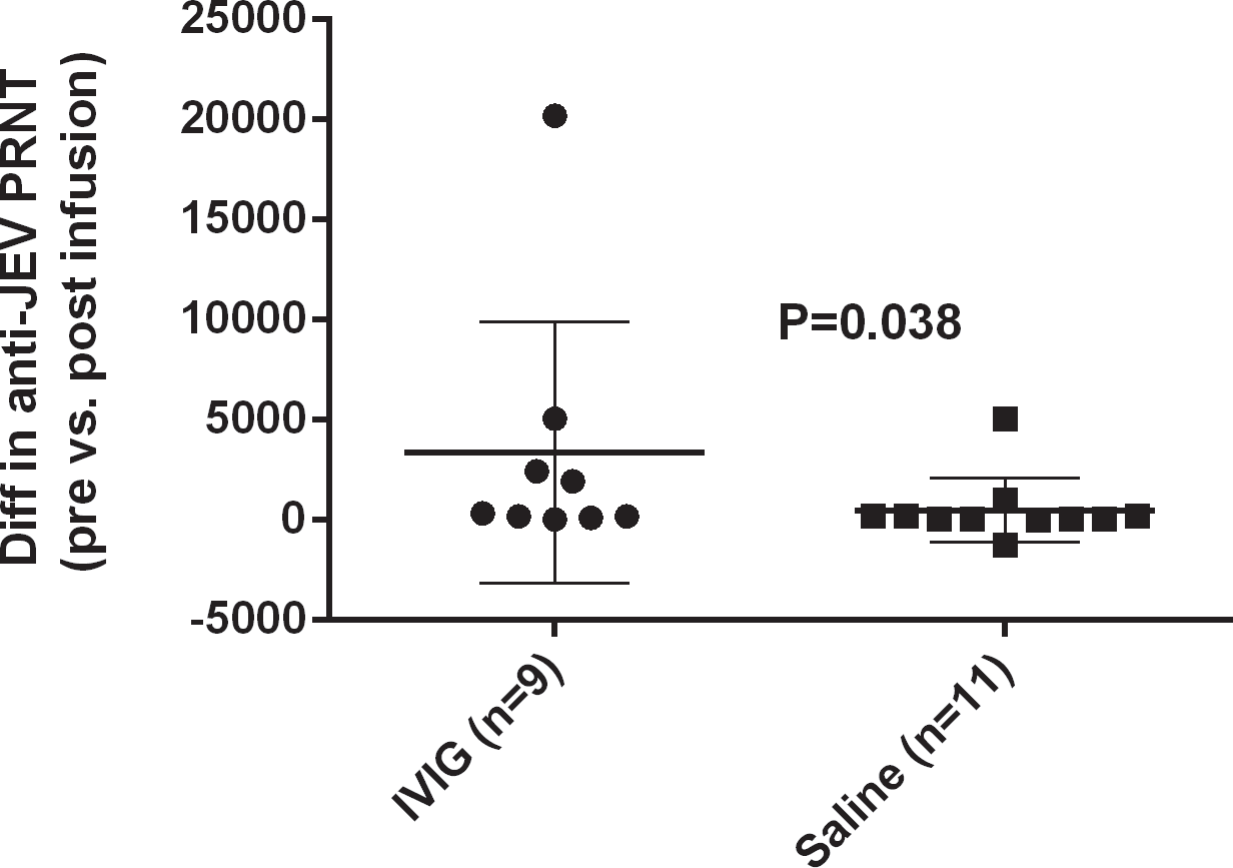
Difference in neutralizing antibody titres to JEV in children with acute encephalitis syndrome treated with IVIG or placebo. Median and inter-quartile range of the difference in JEV PRNT_50_ titres pre and post treatment is presented. Patients are grouped according to treatment. Difference in tires was assessed via Wilcoxon-Mann-Whitney test. Note: Two patients who received IVIG were not included in this analysis because of insufficient sample to undertake PRNT measurements.

The difference in neutralizing antibody titres pre and post treatment were analysed by treatment group (IVIG or placebo) Neutralizing antibody titres increased significantly among participants who received IVIG compared to placebo (p = 0.038: Fig 3). Separating results by anti-JEV IgM antibody status, titres showed a greater increase among those who received IVIG compared to placebo in both anti-JEV antibody status groups. The median increase in neutralizing antibody titre was greater (16 fold) in anti-JEV IgM positive compared to negative patients (median increase in titre post treatment was 1920 versus 120 for IgM positive and negative patients respectively; S4 Table and S1 Fig). However, the increase in neutralizing antibody titres following IVIG treatment was only significant among anti-JEV IgM negative patients (p = 0.048 and p = 0.24 respectively). Patient anti-JEV IgM status prior to treatment was not a significant source of variation in change in neutralizing antibody titre as assessed by 2 way ANOVA (p = 0.29; S5 Table).

### Cytokines

**Fig 4:**
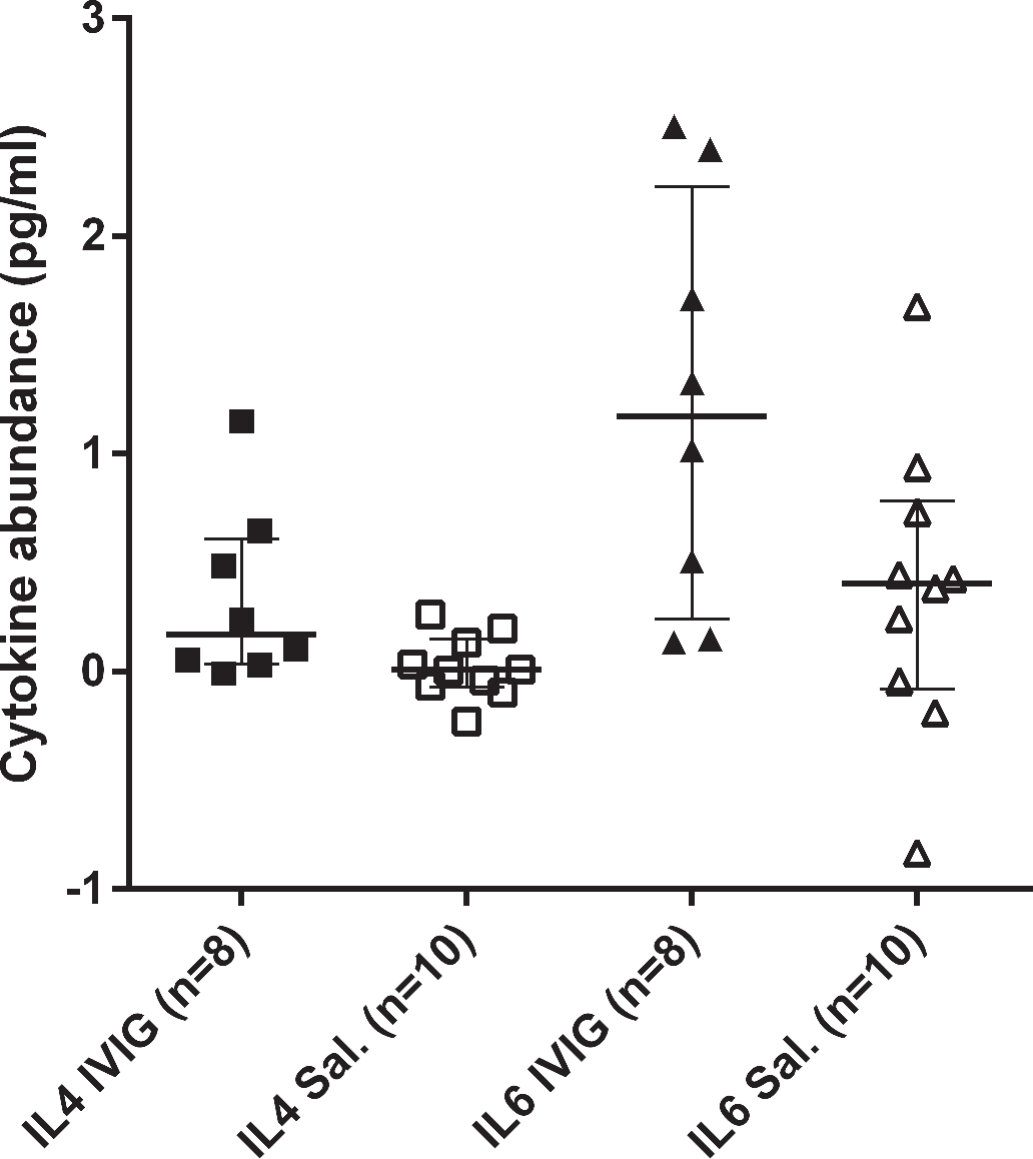
Interleukin (IL)-4 and IL-6 abundance in children with acute encephalitis syndrome receiving intravenous immunoglobulin (IVIG) and placebo. Median and inter-quartile range of change in cytokine abundance (pg/ml) pre and post treatment is presented for IL-4 and IL-6 separately. Cytokine abundance increased for both IL-4 and IL-6. This increase was significant for IL-4 (p = 0.043 and p = 0.068 for IL-4 and IL-6 respectively). Difference in abundance was assessed via Wilcoxon-Mann-Whitney test. Note: Four patients (three who received IVIG and one who received saline) were not included in this analysis because of insufficient sample to undertake the ELISA.

The difference in cytokine abundance pre and post treatment were also analysed by treatment group (Fig 4). Cytokine abundance for both IL-6 and IL-4 increased among participants who received IVIG compared to placebo. This increase was significant for IL-4 (p = 0.043). Separating the IL-4 results by anti-JEV IgM antibody status, IL-4 levels showed a greater increase among those who received IVIG compared to placebo in both anti-JEV antibody status groups. The median increase in IL-4 following IVIG treatment was greater (13 fold) in anti-JEV IgM negative compared to positive patients (median increase in abundance post treatment was 0.65 versus 0.05 pg/ml for IgM negative and positive patients respectively). Neither increase was significant; p = 0.06 and p = 0.6 for IgM negative and positive patients respectively (S6 Table; S7 Table; S2 Fig). Patient anti-JEV IgM status prior to treatment was a significant source of variation in change in IL-4 abundance (p = 0.0018). However, treatment (IVIG or Placebo) was a more significant source of variation as assessed by 2 way ANOVA (p = 0.0005). Patient anti-JEV IgM status prior to treatment was not a significant source of variation in change for IL-6 (S5 Table). A very low concentration of IL-6 was detected in the IVIG (0.4 pg/ml). IL-4 was not detected. Raw data for PRNT levels are available in S8 Table.

## Discussion

JEV, the most important cause of epidemic encephalitis globally, is just one of several important flaviviruses that cause encephalitis. These include tick-borne encephalitis virus, which occurs across northern and eastern Europe, and WNV, which circulates in India, Africa, Europe, and since 1999, the Americas. There is no established treatment for any flavivirus. Although JE vaccine programs are being implemented across Asia, the disease is continuing to spread, and recent estimates put the incidence closer to 70,000 cases annually, rather than the previously estimated 50,000. [34]. JE was first recorded in Nepal in 1978 in Rupandehi district of the Western Development Region [35]. It is currently endemic in 24 districts with sporadic reports from a further 33 of the total 75 districts. Up to 2012 there were 29,877 clinically diagnosed cases with 5,589 deaths (personal communication: Dr Santosh Gurung, Programme for Immunization Preventable Diseases, WHO, Kathmandu, Nepal) In the early outbreaks, the mortality was up to 60%, but more recently this has improved to approximately 20% [30, 35]. Immunization against JE began in 1999 in 3 districts [36, 37], which had been extended to cover 31 high risk districts by 2011. In addition, the Government vaccinated around 200 000 pigs in the Terai zone in 2001 [35]. A recent seroprevalence study in animal hosts in 10 districts in Nepal revealed that 48% of pigs, 27% of ducks and 50% of horses were JEV seropositive demonstrating a high transmission of JEV in the wild and confirming that it is likely to remain [38]. Hence, JE is still a major public health problem in Nepal, and there is a pressing need to develop better treatments.

Our pilot study suggests that a multi-centre RCT of IVIG for children with suspected JE is feasible in this setting with no protocol violations. The double-blinding procedure, using masking tape to cover syringe contents, has been used in a previous study of IFN-α in JE in a very well resourced research unit in Vietnam, which employs research nurses and technicians [23]; we found that it also worked well in this much simpler setting with hospital nurses and technicians. In considering the study, there had been concern that starting an infusion at a low rate, and then increasing over time would be unacceptable to hospital staff if they could not see what drug they were giving; but again this aspect worked well. Although our numbers were small, we did not detect any significant differences in adverse events between the two groups. We did not study the minimum 30 subjects recommended by US FDA Guideline on Safety, Efficacy, and Pharmacokinetic Studies to Support Marketing of Immune Globulin Intravenous (Human) as Replacement Therapy for Primary Humoral Immunodeficiency (2008)(http://www.gmp-compliance.org/guidemgr/files/IGIVIMMUNO.PDF). Nor did we employ a dose escalation designs used in many oncology studies. However we were not studying Primary Humoral Immunodeficiency or cancer, and recommendations from western settings may not always be appropriate for studies in resource-poor settings [39]. Three patients died. The one death in the IVIG group was attributed to severe underlying disease rather than the IVIG, and the two deaths which had occurred by follow-up were both in the placebo group. The proportion of patients exhibiting full recovery (without any sequelae) was similar between the groups at discharge and slightly higher among in the IVIG group at follow. This difference was not significant on intention-to-treat analysis.

In Nepal, IVIG, which has general anti-inflammatory effects, is currently being used to treat a range of pediatric conditions including Guillain-Barré syndrome, idiopathic thrombocytopenic purpura and Kawasaki disease. IVIG from endemic areas is considered a potential treatment for flavivirus encephalitis, including that caused by JEV and WNV, because of the neutralizing antibody it contains [24]. We found IVIG from a range of manufactures in Asia contained significant neutralizing antibody; most had PRNT_50_ titres ≥1:640. Pre-clinical studies suggest that passively transferred antibody may be effective against flavivirus encephalitis [17, 21, 24, 40–46]. Konishi *et al* demonstrated that neutralizing antibody prevents virus dissemination from the peripheral site to the brain, and that antibody-mediated mechanisms of protection were more efficient than cytotoxic T cell responses [43]. In animal studies in which IVIG containing anti-WNV specific antibody was administered during the viraemic phase but before the virus had entered the CNS, there was a dramatic 100% survival rate [24, 44], and mortality was reduced up to five days after infection [45, 46]. More relevant for the treatment of patients presenting to hospital with WN neuroinvasive disease, in which the virus has already entered the CNS, Morrey et al showed that peripheral administration of anti-virus monoclonal antibodies in a mouse model neutralizes WNV even after it has entered the brain [47, 48].

As expected, patients had a greater increase in neutralizing antibody titres among those treated with IVIG compared to placebo. Interestingly JEV antibody positive children treated with IVIG exhibited higher (approximately 16 times higher) titres of neutralizing antibody compared to levels of change following IVIG treatment among JEV antibody negative patients (this change was not significant). The magnitude of this effect appears greater than can be explained by passive transfer of anti-JEV antibody (observed as the increase titres among the JE negative patients). The reason for this is not certain, though it is possible that passively transferred antibody is enhancing the natural production of neutralizing antibody by B cells, perhaps through augmenting the uptake of viral particles by antigen presenting cells. Such a mechanism has been described in a macaque model of HIV, where the administration of low level neutralizing monoclonal antibodies to simian HIV led to the rapid development of neutralizing antibodies through enhanced B cell responses [49]. A relatively modest neutralizing antibody titre (i.e. 1:10) has been shown to protect against JEV in animal models, when antibody is administered prior to infection. However, to our knowledge, no studies have determined what titre is required to limit the evolution of encephalitis once JEV infection is established in humans. Our patients all had an antibody titre that was much higher, around 100 fold higher, than this and yet still had encephalitis.

Antibody-dependent enhancement of viral entry into macrophages is also important in secondary DENV infections. In this case non-neutralising antibody from a prior infection with a different DENV serotype is thought to enhance viral entry into macrophages and contribute to increased disease severity [50]. In tick-borne encephalitis, passive immunization with neutralizing antibody containing IVIG has been used as prophylaxis in those bitten by infected ticks, before disease develops [51]. However, following suspicions that this was actually precipitating disease, through postulated antibody dependent enhancement, this approach was abandoned. Our study suggests there were no significant side effects of treatment with IVIG in JE and administration was associated with increased JEV-specific serum neutralizing antibody titres.

Neuronal cell death in JE may occur directly, from viral cytopathology, and indirectly via immune mediated mechanisms.This may include over activation of microglia cells [52], which release pro-inflammatory cytokines such as interleukin 6 (IL-6), TNF-α, and RANTES (regulated upon activation, normal T cell expressed and secreted), causing massive migration and infiltration of leukocytes into the brain [53]. IL-6, which is produced by neurons, microglia, astrocytes and recruited macrophages in response to viral CNS infection [54–56], causes an increased permeability of the blood brain barrier, which leads to interstitial cerebral edema, and raised intracranial pressure [57, 58]. TNF-α is also produced by astrocytes and macrophages. Its multiple pro-inflammatory properties include upregulation of class I and II MHC expression, upregulation of cellular adhesion molecules, increased permeability of the blood brain barrier [59], and upregulation of inducible nitric oxide synthase (iNOS), leading to the production of nitric oxide (NO) [60]. In addition to affecting virally-infected cells, the inflammatory response in the CNS may also damage non-infected cells to cause bystander cell death.

The elevated IL-6 and IL-4 responses observed in our patients support the hypothesis that administration of IVIG modulates the immune response. Accepting cytokine responses are complex, we chose to measure IL-6 and IL-4 because they have been used previously to give a simple reflection of the balance of pro- to anti- inflammatory responses [61]. IVIG treatment has previously been linked with both reduced and elevated levels of IL-6 among patients [62–68]. Similarly, IVIG treatment has been related to increased IL-4 levels [69, 70]. Both IL-4 and IL-6 participate in the development of antibody responses; IL-4 promotes B cell proliferation and isotype switching and IL-6 induces differentiation of B cells into antibody secreting plasma cells [71]. The modest increase in IL-4 and IL-6 could be consistent with augmentation of an antibody response; therefore the increase in both antibody and pro-antibody producing cytokines may reflect part of the same process [72]. The variation in response among patients with different anti-JEV antibody statuses indicates the immune modulation by IVIG is highly intricate.

Other mechanisms of IVIG induced cytokine production may involve the generation of immune complexes of JEV antigen and IVIG derived antibody. In turn, these complexes may stimulate monocytes to produce IL-6 via Fc-receptor interactions [73]. Other immunomodulatory factors (e.g., sCD4, sCD8, sHLA antigen) present in the IVIG could also induce cytokine production [74, 75].

Because previous studies have shown IL6 is sometimes detected in IVIG [68, 76], we checked the levels in our treatment batches and found that although there were low levels of IL6, this was not enough to explain the elevated titre seen in our treated patients.

When JE is due to primary infection (i.e. first flavivirus infection) a quick and effective IgM response occurs in the serum and CSF within few days, and attempts to isolate virus from either sample are unlikely to be successful. [2] However, absence of IgM is associated with positive virus isolation from the CSF and a bad outcome [2]. When individuals have asymptomatic JEV infection, antibody probably protects the host by restricting viral replication during the viraemic phase, before the virus crosses the blood brain barrier [2]. Antibody may also limit damage during established encephalitis by neutralizing extracellular virus and facilitating lysis of infected cells by antibody-dependent cellular cytotoxicity.

A better understanding of immunological changes in JE and the effects of IVIG will be important for the further development of IVIG treatment [61]. Since this study showed our approach to be feasible, a larger study, with further close monitoring of adverse effects is recommended. The question of efficacy will only be answered with a full phase III randomized placebo-controlled trial.

There may be other wider implications of this study: the number of antibody-mediated encephalitidies being identified is increasing and IVIG is a standard treatment option in these cases [77, 78]. In addition, IVIG may have a role in other viral encephalitis as there is evidence that damage caused is over and above that from direct viral infection alone [18, 79]. In most series of encephalitis patients the aetiology is unknown in the majority of cases, but is presumed to be a mixture of microbial and immune mediated causes [78, 80]; whether IVIG may be a suitable treatment for such patients is unclear. Our study provides important pilot data, but further research is needed in this area.

## Supporting Information

S1 CONSORT Checklist CONSORT checklist. (DOC)

S1 Fig Change in PRNT among treatment participants, sub-grouped by their anti-JEV IgM antibody status. (DOC)

S2 Fig Change in IL-4 abundance among treatment participants, sub-grouped by anti-JEV IgM antibody status. (DOC)

S1 Protocol Trial Protocol. (PDF)

S1 Table Randomization Schedule. (XLS)

S2 Table Participant Characteristics (raw data). (XLS)

S3 Table Adverse events (raw data). (XLS)

S4 Table Change in PRNT titres—pre compared to post treatment. (DOC)

S5 Table ANOVA data based on the linear models for the change in neutralizing antibody titres, IL-4 and IL-6. (DOC)

S6 Table Change in cytokine abundance—pre compared to post treatment. (DOC)

S7 Table Change in IL-4 abundance—pre compared to post treatment, sub-grouped by anti-JEV IgM antibody status. (DOC)

S8 Table PRNT and cytokine levels (raw data). (XLS)
