# Correction: A Preliminary Randomized Double Blind Placebo-Controlled Trial of Intravenous Immunoglobulin for Japanese Encephalitis in Nepal

**DOI:** 10.1371/journal.pone.0136008

**Published:** 2015-08-13

**Authors:** Ajit Rayamajhi, Sam Nightingale, Nisha Keshary Bhatta, Rupa Singh, Elizabeth Ledger, Krishna Prasad Bista, Penny Lewthwaite, Chandeshwar Mahaseth, Lance Turtle, Jaimie Sue Robinson, Sareen Elizabeth Galbraith, Malgorzata Wnek, Barbara Wilmot Johnson, Brian Faragher, Rachel Kneen, Michael John Griffiths, Tom Solomon

Rachel Kneen should be listed as the fifteenth author. Her affiliations are 1: Institute of Infection and Global Health, University of Liverpool, Liverpool, United Kingdom, and 8: Alder Hey Children’s National Health Service Foundation Trust, Liverpool, United Kingdom. The author contributions of this author are as follows: conceived and designed the experiments, performed the experiments, analyzed the data, contributed reagents/materials/analysis tools, wrote the manuscript.
