# Supplementary material for: A Preliminary Randomized Double Blind Placebo-Controlled Trial of Intravenous Immunoglobulin for Japanese Encephalitis in Nepal
Source: PLoS One. 2015 Apr 17;10(4):e0122608. doi: 10.1371/journal.pone.0122608 (PMC4401695; doi:10.1371/journal.pone.0122608)
Supplement: S1 Fig — (DOC) [file pone.0122608.s002.doc]

**Figure S1. Change in PRNT among treatment participants, sub-grouped by their anti-JEV IgM antibody status**

**
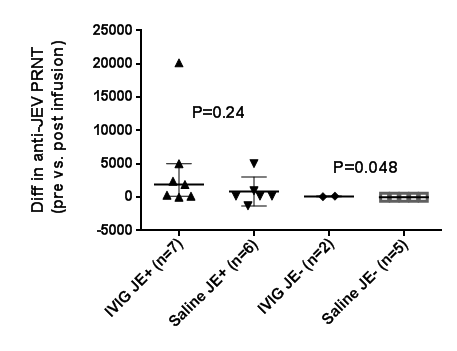
**

**M**edian and inter-quartile range of the difference in plaque reduction neutralising antibody titres (PRNT) against JEV pre and post treatment is presented as four groups. Patients are sub-grouped by treatment exposure (IVIG or Saline) and anti-JEV IgM antibody status prior to treatment (JE+ or JE-).

Titres showed a greater increase among those who received IVIG compared to placebo in both anti-JEV antibody status groups. However, the increase was only significant among anti-JEV IgM negative patients (p=0.048). Differences between subgroups were assessed via Wilcoxon-Mann-Whitney test.

Note: Two patients who received IVIG and were anti-JEV IgM negative were not included in this analysis because of insufficient sample to undertake PRNT measurements.
