# Supplementary material for: A Preliminary Randomized Double Blind Placebo-Controlled Trial of Intravenous Immunoglobulin for Japanese Encephalitis in Nepal
Source: PLoS One. 2015 Apr 17;10(4):e0122608. doi: 10.1371/journal.pone.0122608 (PMC4401695; doi:10.1371/journal.pone.0122608)
Supplement: S2 Fig — (DOC) [file pone.0122608.s003.doc]

**Figure S2. Change in IL-4 abundance among treatment participants, sub-grouped by their anti-JEV IgM antibody status**

**
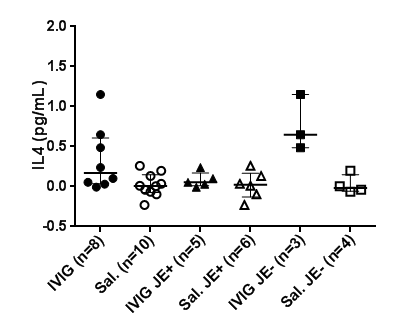
**

**M**edian and inter-quartile range of change in IL-4 abundance (pg/ml) pre and post treatment is presented as six groups. Patients are initially grouped according to treatment. Then they are sub-grouped by both treatment exposure (IVIG or Saline) and anti-JEV IgM antibody status prior to treatment (JE+ or JE-). Overall, IL-4 abundance increased significantly among those who received IVIG compared to saline (p=0.043). There was no significant increase in abundances among JE- or JE + sub-groups (p=0.057 and p=0.65 respectively). Differences in abundance were assessed via Wilcoxon-Mann-Whitney test. Note: Four patients (three who received IVIG and one who received saline) were not included in this analysis because of insufficient sample to undertake the ELISA.
