# Supplementary material for: A Preliminary Randomized Double Blind Placebo-Controlled Trial of Intravenous Immunoglobulin for Japanese Encephalitis in Nepal
Source: PLoS One. 2015 Apr 17;10(4):e0122608. doi: 10.1371/journal.pone.0122608 (PMC4401695; doi:10.1371/journal.pone.0122608)
Supplement: S1 Protocol — (PDF) [file pone.0122608.s004.pdf]

# TRIAL PROTOCOL

## Trial Title: A preliminary randomized double blind placebo-controlled trial of intravenous immunoglobulin for Japanese encephalitis in Nepal

**Sponsor:**
University of Liverpool
**Collaborators:**
Kanti Children's Hospital
B.P. Koirala Institute of Health Sciences
**Information provided by (Responsible Party):** Ajit Rayamajhi, University of Liverpool
ClinicalTrials.gov Identifier:NCT01856205

## Purpose

Japanese encephalitis is caused by a viral infection of the brain transmitted by the bite of an infected mosquito. Patients with Japanese encephalitis can rapidly develop worsening conscious level and seizures. Around a third will die from the infection and half of survivors have serious long-term neurological disability. The majority of those affected are children. There are many causes of viral encephalitis, however Japanese encephalitis virus is the most common cause worldwide with over 60,000 cases annually. It occurs over much of Asia and the geographical range is expanding. There is no specific treatment for Japanese encephalitis virus, although several have been trialed. In this study we examined the effect of a new treatment, called intravenous immunoglobulin, on children with Japanese encephalitis in Nepal. Prior studies have suggested intravenous immunoglobulin may neutralize Japanese encephalitis virus and suppress damaging inflammation in the brain. It has previously been used in individual cases but never examined in a randomized trial. There was recently a trial of IVIG in West Nile encephalitis in the United States, in which Professor Solomon was on the Scientific Advisory Committee. In this study we will look if intravenous immunoglobulin is safe in this context, and that this treatment may alter the way the immune system manages the infection. Therefore, in this pilot study we will test the hypothesis that IVIG can be safely given to children with suspected JE, with no increased risk of serious adverse events compared with placebo. The aim of this proposal is to conduct a pilot safety and tolerability randomized placebo controlled trial of intravenous immunoglobulin (IVIG) in patients with Japanese encephalitis, to explore the relationship between JEV viral load, pro-inflammatory markers called cytokines and blood brain barrier markers, and the effect of IVIG on these relationships.

| Condition | Intervention | Phase |
|---|---|---|
| Japanese Encephalitis | Drug: Intravenous immunoglobulin [ImmunoRel™ (batch 20081217)] | Phase 2 |

| | |
|---|---|
| Study Type: | Interventional |
| Study Design: | Allocation: Randomized<br>Endpoint Classification: Safety/Efficacy Study<br>Intervention Model: Parallel Assignment<br>Masking: Double Blind (Caregiver, Investigator, Outcomes Assessor)<br>Primary Purpose: Treatment |
| Official Title: | A Preliminary Randomized Double Blind Placebo Controlled Trial to Assess the Safety and Efficacy of Intravenous Immunoglobulin (IVIG) in Children With Japanese Encephalitis in Nepal |

**Primary Outcome Measures:**

- Evidence of side effects of study drug such as infusion site reaction, diarrhea, rise in blood pressure and change in urinary output [ Time Frame: Every 12 hours after administration of study drug upto discharge, which is on average eigth day (192 hours) of hospital admission ]

  Patients will be monitored for side effects such as infusion site reaction, diarrhea, rise in blood pressure (in mm Hg) and change in urinary output (in ml/Kg/hour) every 12 hours from first day of commencing treatment until until death or discharge. Patient on average are administered the study drug on the first day of admission. The study drug is administered daily for 5 days. Patients are discharged on an average on eighth day (192 hours) of hospital admission.

**Secondary Outcome Measures:**

- Death or neurological sequelae [ Time Frame: At the time of discharge, an expected average of eighth day of admission and again at 6 months after discharge ]

  At the time of discharge(expected average of eighth day of admission) or death: Time to death, to recover from coma, to sit independently, to stand independently, to walk at least 5m independently, and to leave hospital.

  At 6 months after discharge: history of further seizures, behavioral changes, evidence of recovery of neurological sequelae such as assessment of ability to sit independently, to stand independently, to walk at least 5 meters independently.

**Other Outcome Measures:**

- Serum JEV PRNT50 level (immunological marker) [ Time Frame: Measured at 3 time points: pre-treatment (just before first dose of study drug on first day), mid-treatment (just prior to fourth dose on fourth day) and post-treatment (one hour after fifth dose on fifth day). ]

Pre-treatment was immediately prior to the first dose of study drug on the first day, mid-treatment was immediately prior to the 4th dose and post-treatment was 1hour after administering the fifth dose of the study drug.

| | |
|---|---|
| Enrollment: | 22 |
| Study Start Date: | May 2009 |
| Study Completion Date: | August 2009 |
| Primary Completion Date: | August 2009 (Final data collection date for primary outcome measure) |

| Arms | Assigned Interventions |
|---|---|
| Placebo Comparator: IVIG in JE (JE-positive)<br>We randomly allocated patients to treatment with IVIG or placebo. Children received either saline or intravenous immunoglobulin (IVIG) [ImmunoRel™ (batch 20081217)] at a dose of 400mg/kg/day for 5 days or an equivalent volume of 0.9% normal saline given intravenous at the rate of 0.01 to 0.02 ml/kg body weight/minute. All investigators, care providers and participants were blinded of the study drug. A second sealed envelope was kept with the patient's notes in case a physician urgently needed to know which drug a patient had received. | Drug: Intravenous immunoglobulin [ImmunoRel™ (batch 20081217)]<br>IVIG group received 400mg/kg/day intravenous at the rate of 0.01 to 0.02 ml/kg body weight/minute for 5 days or appearance of side effect or adverse events.<br>Placebo group received 0.9% saline intravenous at similar rate. |
| Placebo Comparator: IVIG in Non-JE(JE-negative)<br>We randomly allocated patients to treatment with IVIG or placebo. Children received either saline or intravenous immunoglobulin (IVIG) [ImmunoRel™ (batch 20081217)] at a dose of 400mg/kg/day for 5 days or an equivalent volume of 0.9% normal saline given intravenous at the rate of 0.01 to 0.02 ml/kg body weight/minute. All investigators, care providers and participants were blinded of the study drug. A second sealed envelope was kept with the patient's notes in case a physician urgently needed to know which drug a patient had received. | Drug: Intravenous immunoglobulin [ImmunoRel™ (batch 20081217)]<br>IVIG group received 400mg/kg/day intravenous at the rate of 0.01 to 0.02 ml/kg body weight/minute for 5 days or appearance of side effect or adverse events.<br>Placebo group received 0.9% saline intravenous at similar rate. |

**Detailed Description:**

Japanese encephalitis (JE) is the most important epidemic encephalitis worldwide, causing approximately 35-50,000 cases and 10-15,000 deaths annually. Half of the survivors have severe neuropsychiatric sequelae, posing a large socio-economic burden on communities that can ill afford it. JE virus (JEV, genus flavivirus, family Flaviviridae) has a 50 nm lipoprotein envelope surrounding a nucleocapsid comprised of core protein and 11 KB of single-stranded positive-sense RNA. The genome has 5' and 3' untranslated regions (UTR), and a single open reading frame encoding genes for three structural proteins (core - C, pre-membrane - prM and envelope - E) and 7 non-structural (NS) proteins. The E protein is critical for viral attachment and entry into cells, and along with NS1 and NS3 is a major target of the immune response.

JEV is an arthropod-borne virus (arbovirus), transmitted in an enzootic cycle between birds, pigs and other vertebrates by mosquitoes, especially Culex species. Almost all of the population in affected parts of Asia is infected by early adulthood, but only a small proportion (about 1 in 300) develops clinical features. These may range from a non-specific febrile illness to a severe meningoencephalitis, in which seizures and clinical signs of raised intracranial pressure are common, and carry a poor prognosis. In addition JEV causes a poliomyelitis-like flaccid paralysis. The distribution of the four genotypes of JEV across Asia was thought to explain the clinical epidemiology, but is now thought to be a reflection of the virus' origin in Southeast Asia, and spread from here. However subtle differences in the genome may be important in determining neurological presentations of flaviviruses.

Although there are vaccines against JEV, they are not available for many of the people that need them, because of issues over cost and production. Progress is being made to overcome these difficulties, and newer vaccines are being used increasingly. In fact Nepal has been at the forefront of the use of a single dose of live attenuated SA14-14-2 vaccine for JE. However, continues to spread across Asia, and there is no treatment. Interferon-α (IFN-α), which is produced as part of the innate response to JEV infection, and which has antiviral activity against the virus, was thought to be the most promising treatment. However, a randomized placebo-controlled trial showed recombinant IFN-α2a at 10 million units/m2/day did not improve the outcome. The pathogenesis of JE is incompletely understood, but by comparison with other positive sense RNA viral encephalitides, is likely to involve a mixture of viral cytopathology and immunopathology .The role of steroids in JEV has been examined in one randomized-placebo controlled trial in Thailand, which failed to show any benefit.

IVIG currently offers one of the best possibilities for improving the outcome in JE. It has been used presumptively in patients with flavivirus encephalitis, including JE, and is postulated to act in two ways: IVIG produced in countries where flaviviruses are endemic contains high titers of specific neutralizing antibody, because most of the population have been exposed to the virus, and thus have antibodies. In addition IVIG has non-specific anti-inflammatory properties, particularly through the suppression of pro-inflammatory cytokines (e.g. in Kawasaki disease). We aim to examine the role of IVIG in JE, and also look at its effects on pro-inflammatory cytokines.

The role of antibody in protection against severe disease in Japanese encephalitis: The humoral immune response in JE has been well characterized. When disease is due to primary infection (i.e. when JEV is the first flavivirus with which an individual has been infected) a rapid and potent IgM response occurs in serum and CSF within days of infection. By day 7 most patients have elevated titers. Attempts to isolate virus are usually negative in such patients. However, the failure to mount an IgM response is associated with positive virus isolation and a fatal outcome.

Antibody to JEV probably protects the host by restricting viral replication during the viraemic phase, before the virus crosses the blood brain barrier. Evidence from other flaviviruses suggests it may also limit damage during established encephalitis by neutralizing extracellular virus and facilitating lysis of infected cells by antibody-dependent cellular cytotoxicity.

In surviving patients immunoglobulin class switching occurs, and within 30 days most have IgG in the serum and CSF. Asymptomatic infection with JEV is also associated with elevated IgM in the serum, but not CSF. In patients with secondary infection (i.e. those who have previously been infected with a different flavivirus, for example dengue infection, or yellow fever vaccination) there is an anamnastic response to flavivirus group common antigens. This secondary pattern of antibody activation is characterized by an early rise in IgG with a subsequent slow rise in IgM.

Inflammation in Japanese encephalitis: At autopsy in JE, the brain is usually edematous, with congestion of the parenchyma . Cerebellar and uncal herniation are common. Histopathologically JE is characterized by perivascular inflammation with recruitment of macrophages, neutrophils and lymphocytes. The basal ganglia and anterior horns cells of the spinal cord are particularly affected, providing a pathological correlate for the Parkinsonism, and poliomyelitis-like flaccid paralysis, which may be seen. Viral antigen is predominantly in neurons although microglial cells, astrocytes and vascular endothelial cells are also infected. When survival is prolonged beyond 7 days acellular necrotic zones are seen, usually in the region of small or medium sized arteries which are surrounded by edema.

Immunopathology in Japanese encephalitis: The pathogenesis of JE is incompletely understood, but available evidence indicates that, as with a number of other positive sense RNA viral encephalitides including lymohoctyic chorio meningitis virus (LCMV) in mice Sindbis virus, dengue virus, yellow fever virus and West Nile virus, there is immune-mediated damage as well as viral cytopathology.

Pro-inflammatory cytokine responses in Japanese encephalitis: As detailed below under Preliminary Studies, there is accumulating evidence that the pro-inflammatory cytokine response may make an important contribution to immunopathology in JE. Among cytokines studied, interleukin (IL)-6, IL-8 chemokine (CXC motif) ligand (CXCL-8), and tumor necrosis factor (TNF)- α appear to be associated with a fatal outcome. There is also new evidence on the importance of other cytokines and chemokines in encephalitis caused by other flaviviruses, particularly West Nile virus. There are several mechanisms by which the pro-inflammatory response may be deleterious. Recent studies have shown IL-6, which is produced by neurons, microglia, astrocytes and recruited macrophages in response to viral CNS infection, causes an increased permeability of the blood brain barrier (BBB), which leads to interstitial cerebral edema, and raised intracranial pressure. TNF-α is produced by microglia, astrocytes and macrophages. Its multiple pro-inflammatory properties include upregulation of class I and II MHC expression, upregulation of cellular adhesion molecules, increased permeability of the BBB, and upregulation of inducible nitric oxide synthase (iNOS), leading to the production of nitric oxide (NO). At high concentrations NO is directly toxic to CNS cells, causing oxidative damage, and apoptosis. Recent evidence suggests that the early cytokine and chemokines responses may also be important in determining whether the virus even gains entry across the blood brain barrier to enter the CNS. In one study of West Nile virus infection, mice deficient in Toll-like receptor 3(Tlr3), which recognizes viral double stranded RNA, were relatively resistant to lethal infection. This was associated with reduced cytokine production (particularly TNF-α and IL-6), and increased viral load in the periphery, compared to wild type mice. TNF-α receptor

1 signaling was found to be vital for blood brain barrier compromise upon Tlr3 stimulation by the virus. In addition to affecting virally-infected cells, the inflammatory response in the CNS may also damage non-infected cells to cause bystander cell death. The importance of the chemokine receptor CCR5, and its ligand CCL5 (also known as RANTES, regulated on activation, normally T cell expressed and secreted) in CNS inflammation caused by flaviviruses was shown recently by their prominent up-regulation in a mouse model of West Nile virus infection. This was associated with CNS infiltration of CD4+ and CD8+ T cells, Natural killer (NK)1.1+ cells and macrophages expressing the receptor. The significance of CCR5 in pathogenesis was established by mortality studies in which infection of CCR5-/- mice was rapidly and uniformly fatal. Importantly, our recent study (see below) found that CCL5 was also elevated in the plasma of humans with JE, where higher levels were associated with a fatal outcome.

Although clinical studies in humans with JE, and parallel studies in animal models of flavivirus encephalitis suggest elevated pro-inflammatory cytokines appear to be important and associated with a bad outcome in JE, it is not clear whether control of this strong pro-inflammatory response will improve the outcome. We will conduct a pilot double-blind placebo-controlled study on safety, tolerability and immunological markers of efficacy of IVIG in children with JE in Nepal, followed by a larger efficacy study.

Preliminary studies: Professor Solomon has been studying JE and related flaviviruses since 1994. With the support of his group and collaborators, he has shown that JEV is the most important cause of viral encephalitis in Vietnam, as it is in most of South and Southeast Asia. He showed that seizures and raised intracranial pressure are common clinical manifestations of infection. In collaboration with colleagues in Malaysia he developed and field-tested a simple rapid diagnostic test for diagnosing JE in the rural settings where it occurs. This test was also able to distinguish JE from dengue - a related flavivirus that circulates in Asia. Although dengue is better known as a cause of hemorrhagic disease, Prof Solomon and his team showed that it is also an important cause of neurological disease. Prof Solomon also showed that as well as presenting with encephalitis, JEV can present with a poliomyelitis-like acute flaccid paralysis, which has also more recently been recognized in adults infected with West Nile virus. Based on in vitro and in vivo data, together with phase I/II studies in humans, Prof Solomon conducted a randomized placebo controlled trial of interferon α-2b in children with JE (the only antiviral trial ever conducted for JE). However the study showed that it did not improve the outcome. To begin exploring the possible contribution of viral genetic diversity to the clinical epidemiology of JE, Prof Solomon examined the molecular evolution of the virus across Asia, and showed that the virus probably originated in the Indonesia and Malaysia region, and evolved here into the different genotypes, the most recent of which subsequently spread across Asia.

The Liverpool group has more recently begun examining inflammation in JE. A recent pathological study in humans and a mouse model confirms the importance of the inflammatory response, and suggests there may be damage to the vascular endothelium. A study of pro-inflammatory and anti-inflammatory cytokines in humans with JE has shown that CSF levels of interleukin(IL)-6, tumor necrosis factor (TNF)-α, interferon (IFN)-α and the chemokine CXCL8 (IL-8) were higher in patients who died than in those who survived. IFN-γ and nitric oxide (NO) were also detected in the CSF of fatal cases and survivors. These findings extend the observations of other investigators on TNF-α and CXCL8 indicating a strong pro-inflammatory response in JE is associated with a poor outcome. It was also shown that CCL5 (RANTES) is

expressed in the plasma of humans with JE, and that high plasma levels are associated with fatal disease. Clinical data from other flavivirus encephalitides support the concept that inflammation may contribute to the pathogenesis. For example, immunosuppressed transplant patients infected with West Nile virus develop CNS disease later than immunocompetent patients. Cytokines do not act in isolation, and indeed the pluripotency and redundancy of biological response is one of their characteristic features. Recent microarray experiments show a wide range of molecules are upregulated in CNS inflammatory conditions. In addition the Liverpool group has studied the relationship between antibody responses and outcome. Along with other investigators, we have shown that the presence of antibody in the serum and CSF is associated with survival.

JE in Nepal: Japanese encephalitis was first recorded in Nepal in 1978 and is currently endemic in 24 districts. Since its first appearance there have been over 26,667 cases and 5381 deaths. The early mortality figures were quoted at up to 60%, but more recently they have improved to approximately 20% in those under 15 years. The morbidity is thought to be around 50%. As a result of concern about the disease, there was immunization against JE in 1999 in 3 districts, this has now been extended to cover the majority of the affected districts. In addition the Government vaccinated around 200 000 pigs in the Terai zone in 2001. Despite these successes there are still ongoing cases of JE. In 2006 there were 292 confirmed cases and 1481 AES cases of unknown etiology. These figures may under represent the actual numbers of cases due to logistical issues, deaths before hospital admission, and difficulties in confirming the diagnosis of JE with paired sera and CSF samples. Recent serosurveillance in the animal hosts for JE in Nepal clearly shows the virus is still present in the wild and is likely to remain. Seroprevalence was 48% (102/212), in pigs, 27% (15/56) in ducks and 50% (6/12) in horses from a total of 280 sera collected from 10 districts of which 44% were positive overall. Hence, JE is still a major public health problem in Nepal, and there is a pressing need to develop better treatments. Currently IVIG is used in Nepal to treat a range of pediatric conditions, including Guillain-Barré syndrome, idiopathic thrombocytopenic purpura.

Rationale for use of IVIG in JE: There is currently no antiviral or immunomodulatory treatment for JE or indeed any flavivirus encephalitis. As outlined above, the only trials conducted, using interferon alpha, and using dexamethasone, failed to show any benefit. Based on data in preclinical studies, and observational studies in humans (see below), IVIG currently offers the best hope for treatment, through its antiviral and anti-inflammatory properties.

Established pediatric uses of IVIG: IVIG has been established as the standard treatment for a number of childhood immune mediated diseases including Guillain-Barré syndrome, Kawasaki's disease, immune thrombocytopenia and Dermatomyositis. In Kawasaki disease, T cell and B cell activation is down regulated by IVIG. In thrombocytopenic purpura blockade of FCγ receptors is thought to be important, which may result in down regulation of secretory cytokines. In Guillain-Barré syndrome and dermatomyositis inhibition of complement binding and prevention of membranolytic attack complex formation are possible mechanisms of IVIG action. In addition, IVIG has been employed in the treatment and prophylaxis of a number of childhood viral infections where the neutralizing antibodies in the IVIG were thought to be important. IVIG has been used to treat, and later to provide prophylaxis, for children with hypo- or agammaglobulinamia with chronic enteroviral meningoencephalitis. Importantly, children with X-linked agammaglobulinaemia and with enteroviral meningoencephalitis treated with intensive and prolonged IVIG therapy have achieved long-lasting clinical and viral remission (negative CSF viral culture and PCR). However, the patients relapsed when IVIG was tapered, indicating a

major beneficial role of IVIG in this condition. IVIG infusion in infants and children with AIDS treated with zidovudine therapy has been shown to reduce the risk of serious bacterial infections (16.9% IVIG group vs 24.3% placebo group (relative risk, 0.60; 95 percent confidence interval, 0.35 to 1.04; p = 0.07). In respiratory syncytial virus (RSV) infections immunoglobulin therapy has been shown to be safe and generally well tolerated in a randomized controlled trial (RCT) involving 102 previously healthy children, though the beneficial effect of reducing hospitalization and intensive care unit day were marginal in children with severe disease. Other established indications for passive immunization with IVIG include parvovirus B19 infection.

Use of IVIG in flavivirus encephalitis: There is strong evidence to suggest that JEV, like other neurotropic flaviviruses, may be more susceptible to antibody-mediated, rather than cell-mediated immune responses. For JEV, and other neurotropic flaviviruses, clearance of virus is not dependent on cytolytic T cell activity, in contrast to non-neurotropic viruses. Neurons, as terminally differentiated cells, do not express MHC-1, which would subject them to lysis by CD8 T cells and non-replacement. Animal data support the importance of antibody mediated immunity. In one study, Konishi et. al. immunized mice with plasmid DNAs encoding JEV proteins that induce neutralizing antibody responses or cytotoxic T lymphocyte responses, and then challenged with lethal intraperitoneal doses of virus. They showed that neutralizing antibody prevents virus dissemination from the peripheral site to the brain, and that antibody-mediated mechanisms of protection were more efficient than the cytotoxic T cell responses. These findings supported earlier work showing that anti-envelope protein antibodies, are the most critical protective component in a JEV challenge model, and more recent passive antibody transfer experiments. In vitro work in mice shows a protective role for IVIG given prophylactically to prevent the flavivirus causing tick borne encephalitis and a protective effect when used as a treatment. In work by several different groups, use of IVIG containing specific anti West Nile Virus antibody during the viraemic phase, before the virus had entered the CNS, showed a dramatic 100% survival. Similar work with Tick borne encephalitis had a 100% effect on survival. There are now good data that in animal models of flavivirus encephalitis, peripheral administration of antibody neutralizes virus even after it has entered the central nervous system.

Clinical Data: Because of the of preclinical data supporting a role for antibody treatment in flavivirus encephalitis, antibody treatment (in the form of IVIG) has also been used on a compassionate basis in JE, and West Nile encephalitis. In addition IVIG is currently being investigated for West Nile encephalitis in a National Institutes of Health (NIH) sponsored randomized placebo controlled trial in the United States (for which Prof Solomon is on the Scientific Steering Committee).

IVIG was used in a 49 year old traveler returning from Vietnam who presented with JE to an Italian hospital. IVIG has also been used to treat West Nile virus infections in five patients in Israel and three in the United States. IVIG was also assessed in a placebo controlled trial for West Nile virus infection in the United States with no serious adverse effects of the drug.

Rationale for Dose: The most commonly used dose of IVIG is 2g/kg, either given as a single infusion, or divided as 400mg/kg for five days. In Kawasaki disease 2g/kg as a single ten hour infusion is well tolerated, and was shown to be the most effective regime. In Guillain-Barré syndrome in children and adults the drug is most often given over 5 days, and this is the regime that has been used in patients with flavivirus encephalitis, and will be used in this study.

Drug and Intervention: Children that meet the entry criteria will be randomly assigned to receive IVIG, (Reliance Biophamaceuticals Pvt. Ltd.) at a dose of 400mg/kg/day for 5 days, or an equivalent volume of 0.9% normal saline, using an established technique for giving fluid treatment in a double blind manner.

Drug Presentation: We will use Immunorel™ intravenous immunoglobulin, which is produced by Reliance Biophamaceuticals Pvt. Ltd., and manufactured in China and is available through the Yetichem Pharmacy, Sundhara, Kathmandu and Shriran Pharmacy, Maharajgunj, Kathmandu. This is the IVIG product currently used most often in Nepal.

As for most other parts of Asia, the seroprevalence of JE in China is high. By age 10, approximately one third of children have neutralizing antibodies to JEV, and by adulthood, the majority of the population has been exposed. There are extensive data showing that IVIG from areas where flaviviruses are endemic contains significant amounts of neutralizing antibody. For example for IVIG from Israel contains moderate to high levels of neutralizing antibody to West Nile Virus. Even though West Nile virus has only circulated in the United States for a few years, high levels of neutralizing antibody have been found in some IVIG lots.

Anti-JEV neutralizing antibody in IVIG from Asia: We have recently examined the neutralizing antibody titres in a range of IVIG products from India and China, using the plaque reduction neutralization assay (PRNT)50. In this study we found that IVIG from the UK, contained no antibody, and serum from a laboratory worker who had been vaccinated against JEV contained a small amount of neutralizing antibody. In contrast IVIG manufactured by Bharrat, Hualan, Sichun and Reliance companies all had neutralizing antibody titres of greater than 1 in 400, with Reliance having the greatest titre, at nearly 1 in 750.

Immunorel™ is packed in clear and colorless vials and comes in 2 pack sizes, each containing IVIG at a dose of 0.05g/ml (ie 50mg/ml). The available vials are, 50ml (containing 2.5g), and 100ml (5.0g).

Children will thus receive 400mg/kg/day of IVIG which is equivalent to 8mls/kg/day of Immunorel™ solution, or 8ml/kg/day of 0.9% normal saline.

Pharmacokinetics: Distribution studies have been done with a range of IVIG preparations. In a study of patients who developed aseptic meningitis (as a complication of the infusion), after receiving 2 g/kg intravenously in 2 divided doses, the serum level increases fivefold and then declines by 50% over the next 72 hours as extravascular redistribution took place. By 21- 28 days, pretreatment levels were reached. The half-life was 18-32 days, and was similar to native immunoglobulin. In the first 48 hours after the infusion the CSF concentration increased 1.5 to 7-fold but returned to normal within a week. CNS penetration may be expected in patients with viral meningoencephalitis or even encephalomyelitis as there will be a break down of the blood-brain-barrier. Metabolism of IVIG takes place in the cells of the reticuloendothelial system where immunoglobulins and immunoglobulin complexes are broken down.

Studies with Immunorel® in normal subjects showed that peak serum concentrations occur immediately after intravenous injection, and are dose related. Within 24 hours up to 30% of a dose may be removed by catabolism and distribution. Data concerning distribution suggest that IVIg distributes throughout intravascular (60%) and extravascular (40%) spaces. The serum half life of immunoglobulin ranges between 21 and 29 days.

Reaction Associated with Intravenous Immunoglobulin: Reactions to intravenous immunoglobulin tend to be related to the infusion rate and are most likely to occur during the first hour of the infusion. We will monitor the patient's vital signs and general status regularly throughout the infusion. The types of reactions that have been reported include abdominal pain, headache, chest-tightness, facial flushing or pallor, hot sensations, dyspnoea, non-urticarial skin rash, itching, hypotension, nausea or vomiting. Should any of these reactions develop during infusion of IVIG, the infusion will be temporarily stopped until the patient improves clinically (5 to 10 minutes) and then cautiously recommenced at a slower rate. Some patients may develop delayed adverse reactions to IVIG such as nausea, vomiting, chest pain, rigors, dizziness or aching legs. These adverse reactions occur after the infusion has stopped but usually within 24 hours. True hypersensitivity reactions to IVIG such as urticaria, angioedema, bronchospasm or hypotension occur very rarely. Should an anaphylactic reaction develop after IVIG, the infusion will be stopped and treatment instituted with adrenaline, oxygen, antihistamine and steroids. Hemolytic anemia and neutropenia have been reported in rare instances in association with IVIG treatment. Mild and moderate elevations of serum transaminases (AST, ALT, gamma GT) have been observed in a small number of patients given IVIG. Such changes were transient and not associated with the transmission of hepatitis. Rare complications an aseptic meningitis syndrome (AMS), thrombophlebitis, renal dysfunction and acute renal failure have occurred in patients receiving IVIG. Liver and renal function will be monitored during the study.

Reactions Associated with Immunorel™:

i. Primary Immune Deficiency: In patients with immunodeficiency syndrome receiving IVIG at a monthly dose of 400 mg/kg body weight, the reactions reported have been malaise, feeling of faintness, fever, chills, headache, nausea, vomiting, chest tightness, dyspnoea and chest, back or hip pain. Mild erythema at the infusion site has also been reported in some cases.

ii. Idiopathic Thrombocytopenic Purpura (ITP): In the treatment of adult and paediatric patients with ITP at a dose of 400 mg/kg body weight, the systemic reactions were observed only in less than 3% of the patients. The other symptoms which were all mild and transient include chest tightness, a sense of tachycardia and a burning sensation in the head. At a dose of 1000 mg/kg body weight either as a single dose or as two doses on consecutive days in the treatment of adult and paediatric patients with ITP. Adverse reactions have been noted only in less than 10% of the patients.

iii. Bone Marrow Transplantation: At a dose of 500 mg/kg body weight 7 days and two days before transplant and weekly through day 90 post-transplant, adverse reactions were reported in less than 7% of the patients. All reactions were classified as mild which include headache, flushing, fever and slight back discomfort.

iv. Renal: Increases in creatinine and blood urea nitrogen (BUN) may be seen as soon as one to two days after an infusion. Progression to oliguria or anuria may require dialysis. Severe occasional adverse events have been reported following IVIG therapy include: acute renal failure, acute tubular necrosis, proximal tubular nephropathy, and osmotic nephosis. Correction of volume depletion by using appropriate fluids prior to initiation of IVIG therapy is therefore essential. Measurement of blood urea nitrogen (BUN) and /or serum creatinine should be performed prior to initial infusion of IVIG and again at appropriate intervals afterwards, with monitoring of urine output.

iv. General: Reactions to IVIG are related to the rate of infusion. Very rarely an anaphylactoid reactions may occur in patients with no prior history of severe allergic reactions to either intramuscular or intravenous immunoglobulin.

Mutagenicity, Carcinogenicity and Impairment of Fertility: Animal reproduction studies have not been conducted with Gamma IV™. Hence it is not known whether IVIG can cause fetal harm when administered to pregnant woman or can affect the reproduction capacity.

Interactions with Other Drugs: The interaction of IVIG with other drugs has not been established in appropriate studies. Passively acquired antibody can interfere with the response to live, attenuated vaccines. Therefore, administration of such vaccines, e.g. poliomyelitis or measles, will be deferred until approximately six months after IVIG infusion. By the same token, immunoglobulins should not be administered for at least two weeks after a vaccine has been given.

Contraindications: IVIG is contraindicated in individuals who are known to have an anaphylactic or severe systemic response to immune Globulin (Human). Individuals with selective lgA deficiencies should not receive Gamma IV™, since these individuals may experience server reactions to the lgA which may be present.

Safety Warnings: The product should not be used if it is turbid. Solutions which have been frozen should not be used. Once opened, even if only partially used, vials should be discarded. IVIG should be administered only intravenously as the intramuscular and subcutaneous routes have not been evaluated.

Dosage and Administration: In general it is recommended that IVIG be administered by itself on an initial rate of 0.01 to 0.02 ml/kg body weighty/minute for 30 minutes, if well tolerated the rate may be gradually increased to a maximum of 0.08 ml/kg body weight/minute. IVIG is recommended to be given by a separate line by itself without mixing with other intravenous fluids or medications the patients might be receiving. IVIG is not compatible with saline. The dilution if required, IVIG may be diluted with 5% Dextrose in water.

**Eligibility**

| | |
|---|---|
| Ages Eligible for Study: | 1 Year to 14 Years |
| Genders Eligible for Study: | Both |
| Accepts Healthy Volunteers: | No |

**Inclusion Criteria:**

- Children aged between 1 and 14 years who had clinically diagnosed encephalitis on the basis of history of fever that lasted less than 14 days, altered consciousness with or without a history of new onset seizures with CSF finding of white cell count less than 1000 cells/mm3 with no organisms on Gram stain and a CSF: plasma glucose ratio > 40% admitted in Kanti Children's Hospital and BP Koirala Institute of Health Sciences, Nepal.

**Exclusion Criteria:**

- Asexual Plasmodium falciparum parasites in blood

- Coma appears secondary to other systemic condition, eg hepatic failure, cardiac failure, toxins.
- Patients who have documented antibiotic treatment before admission and in whom partially treated bacterial meningitis appears more likely than encephalitis
- Children with simple febrile convulsions, defined as a single seizure lasting less than 15 minutes followed by full recovery of consciousness within 60 minutes.
- Pregnant or breastfeeding females
- Children with a GCS of 3/15, who were receiving artificial ventilation without signs of spontaneous respiration, and with absent oculocephalic reflex.
