# Supplementary material for: A Preliminary Randomized Double Blind Placebo-Controlled Trial of Intravenous Immunoglobulin for Japanese Encephalitis in Nepal
Source: PLoS One. 2015 Apr 17;10(4):e0122608. doi: 10.1371/journal.pone.0122608 (PMC4401695; doi:10.1371/journal.pone.0122608)
Supplement: S4 Table — (DOC) [file pone.0122608.s008.doc]

**Table S4. Change in PRNT titres - pre compared to post treatment.**

|  | **IVIG (n=11)** | | | **Placebo (n=11)** | | |
| --- | --- | --- | --- | --- | --- | --- |
|
|  | **1. Total** | **2. JE +ve** | **3. JE -ve** | **4. Total** | **5. JE +ve** | **6. JE -ve** |
| Number | 9 | 7 | 2 | 11 | 6 | 5 |
| Minimum | 0 | 0 | 80 | -1280 | -1280 | -10 |
| 25% Percentile | 120 | 160 | 80 | 0 | -207.5 | -5 |
| Median | 320 | 1920 | 120 | 0 | 160 | 0 |
| 75% Percentile | 3720 | 5040 | 160 | 160 | 1980 | 0 |
| Maximum | 20160 | 20160 | 160 | 5040 | 5040 | 0 |
| Lower 95% CI | 80 | 0 | 80 | -10 | -1280 | 80 |
| Upper 95% CI | 5040 | 20160 | 5040 | 960 | 5040 | 160 |
| P val. Grp. 1 vs. 4 | 0.038 |  |  |  |  |  |
| P val. Grp. 2 vs. 5 |  | 0.244 |  |  |  |  |
| P val. Grp. 3 vs. 6 |  |  | 0.048 |  |  |  |

The table presents change in PRNT titres (pre versus post treatment) among treatment groups. Patients are sub-grouped by their anti-JEV IgM antibody status prior to treatment (JE+ or JE-).

Total - indicates number of patients where PRNT titres were available pre and post treatment.

Confidence intervals (CI) represent estimated 95% limits around the median.

There was a markedly higher increase in PRNT titres following IVIG treatment among anti-JEV antibody positive compared negative patients (16 x higher). This difference was not statistically significant. Negative PRNT values indicate a fall in titres following treatment.

P values calculated by Wilcoxon-Mann-Whitney test.

Note: Two patients, who received IVIG and were JE antibody negative, did not have sufficient sample to undertake PRNT measurement.
