# Supplementary material for: A Preliminary Randomized Double Blind Placebo-Controlled Trial of Intravenous Immunoglobulin for Japanese Encephalitis in Nepal
Source: PLoS One. 2015 Apr 17;10(4):e0122608. doi: 10.1371/journal.pone.0122608 (PMC4401695; doi:10.1371/journal.pone.0122608)
Supplement: S5 Table — (DOC) [file pone.0122608.s009.doc]

**Table S5.** ANOVA data based on the linear models for the change in neutralizing antibody titres IL-4 and IL-6.

| **Two-way ANOVA** |  |  |
| --- | --- | --- |
| **Source of Variation** | **% of total variation** | **P value** |
| **Interaction for PRNT titres** | 2.65 | 0.4843 |
| JE status (Anti-JEV IgM + or -) | 6.168 | 0.2908 |
| Treatment (IVIG or Sal.) | 3.057 | 0.4531 |
|  |  |  |
| **Interaction for IL-4** | 26.86 | 0.002 |
| JE status (Anti-JEV IgM + or -) | 27.77 | 0.0018 |
| Treatment (IVIG or Sal.) | 38.16 | 0.0005 |
|  |  |  |
| **Interaction for IL-6** | 0.01091 | 0.9667 |
| JE status (Anti-JEV IgM + or -) | 0.4206 | 0.7954 |
| Treatment (IVIG or Sal.) | 7.871 | 0.2715 |

The table presents source of variation for change in PRNT, IL-4 and IL-6 abundance.

The table shows the interaction between participants’ anti-JEV IgM antibody status and Treatment group

The data was calculated via Two-way ANOVA.
