# Supplementary material for: A Preliminary Randomized Double Blind Placebo-Controlled Trial of Intravenous Immunoglobulin for Japanese Encephalitis in Nepal
Source: PLoS One. 2015 Apr 17;10(4):e0122608. doi: 10.1371/journal.pone.0122608 (PMC4401695; doi:10.1371/journal.pone.0122608)
Supplement: S6 Table — (DOC) [file pone.0122608.s010.doc]

**Table S6. Change in cytokine abundance - pre compared to post treatment.**

|  | **IL-4** | |  | **IL-6** | |
| --- | --- | --- | --- | --- | --- |
|  | **IVIG** | **Placebo** |  | **IVIG** | **Placebo** |
| Number | 8 | 10 |  | 8 | 10 |
| Minimum | -0.01 | -0.23 |  | 0.13 | -0.83 |
| 25% Percentile | 0.04 | -0.07 |  | 0.24 | -0.08 |
| Median | 0.17 | 0.01 |  | 1.17 | 0.4 |
| 75% Percentile | 0.61 | 0.15 |  | 2.23 | 0.78 |
| Maximum | 1.15 | 0.26 |  | 2.51 | 1.68 |
| Lower 95% CI | -0.007 | -0.096 |  | 0.133 | -0.189 |
| Upper 95% CI | 1.151 | 0.197 |  | 2.506 | 0.938 |
| P val.  IVIG vs. placebo | 0.04 |  |  | 0.067 |  |

The table presents change in cytokine abundance (pg/ml) pre versus post treatment among treatment groups for IL-4 and IL-6.

Number - indicates number of patients where cytokine abundance measurements were available pre and post treatment. Negative values indicate a fall in abundance following treatment.

Confidence intervals (CI) represent estimated 95% limits around the median.

P values calculated via Wilcoxon-Mann-Whitney test.

Note: Four patients, three who received IVIG and one who received placebo, did not have sufficient sample to undertake cytokine measurements.
