# Supplementary material for: A Preliminary Randomized Double Blind Placebo-Controlled Trial of Intravenous Immunoglobulin for Japanese Encephalitis in Nepal
Source: PLoS One. 2015 Apr 17;10(4):e0122608. doi: 10.1371/journal.pone.0122608 (PMC4401695; doi:10.1371/journal.pone.0122608)
Supplement: S7 Table — (DOC) [file pone.0122608.s011.doc]

**Table S7. Change in IL-4 abundance - pre compared to post treatment, sub-grouped by anti-JEV antibody status**

|  | **Total IL-4** | | **IL-4 JE+** | | **IL-4 JE-** | |
| --- | --- | --- | --- | --- | --- | --- |
|  | **IVIG (n=8)** | **Sal. (n=10)** | **IVIG (n=5)** | **Sal. (n=6)** | **IVIG (n=3)** | **Sal. (n=4)** |
| Number | 8 | 10 | 5 | 6 | 3 | 4 |
| Minimum | -0.01 | -0.23 | -0.01 | -0.23 | 0.49 | -0.07 |
| 25% Percentile | 0.04 | -0.07 | 0.01 | -0.13 | 0.49 | -0.06 |
| Median | 0.17 | 0.01 | 0.05 | 0.02 | 0.65 | -0.02 |
| 75% Percentile | 0.61 | 0.15 | 0.17 | 0.16 | 1.15 | 0.15 |
| Maximum | 1.15 | 0.26 | 0.24 | 0.26 | 1.15 | 0.2 |
| Lower 95% CI | -0.007 | -0.096 | -0.007 | -0.229 | 0.486 | -0.065 |
| Upper 95% CI | 1.151 | 0.197 | 0.237 | 0.26 | 1.151 | 0.197 |
| P val. IVIG vs. Saline | 0.043 |  | 0.649 |  | 0.057 |  |

The table presents change in IL-4 abundance (pg/ml) pre versus post treatment among treatment groups (IVIG or saline). Patients are sub-grouped by their anti-JEV IgM antibody status prior to treatment (JE + or JE-). Number - indicates number of patients where cytokine abundance measurements were available pre and post treatment.

Confidence intervals (CI) represent estimate of 95% limits around the median.

P values calculated via Wilcoxon-Mann-Whitney test.

The median increase in IL-4 following IVIG treatment was greater (13 fold) in JE- compared to JE+ patients (median increase in abundance was 0.65 versus 0.05 pg/ml respectively).

Note: Four patients, three who received IVIG and one who received saline, did not have sufficient sample to undertake cytokine measurements.
